# Synthesis, Spectroscopic, Structural and Molecular Docking Studies of Some New Nano-Sized Ferrocene-Based Imine Chelates as Antimicrobial and Anticancer Agents

**DOI:** 10.3390/ma15103678

**Published:** 2022-05-20

**Authors:** Mai M. Khalaf, Hany M. Abd El-Lateef, Abdulrahman Alhadhrami, Fatma N. Sayed, Gehad G. Mohamed, Mohamed Gouda, Saad Shaaban, Ahmed M. Abu-Dief

**Affiliations:** 1 Department of Chemistry, College of Science, King Faisal University, Al-Ahsa 31982, Saudi Arabia; hmahmed@kfu.edu.sa (H.M.A.E.-L.); mgoudaam@kfu.edu.sa (M.G.); sibrahim@kfu.edu.sa (S.S.); 2 Department of Chemistry, Faculty of Science, Sohag University, Sohag 82534, Egypt; 3 Department of Chemistry, Colleague of Science, Taif University, P.O. Box 11099, Taif 21944, Saudi Arabia; babatin2002@hotmail.com; 4 Chemistry Department, Faculty of Science, Cairo University, Giza 12613, Egypt; fatmanabilsayed85@gmail.com (F.N.S.); ggenidymohamed@sci.cu.edu.eg (G.G.M.); 5 Nanoscience Department, Basic and Applied Sciences Institute, Egypt-Japan University of Science and Technology, New Borg El Arab, Alexandria 21934, Egypt; 6 Chemistry Department, Faculty of Science, Mansoura University, Mansoura 35516, Egypt; 7 Department of Chemistry, College of Science, Taibah University, P.O. Box 344, Madinah 42353, Saudi Arabia

**Keywords:** organometallic acetyl ferrocene imine ligand, nanomaterials, 2-aminothiophenol, DFT, antimicrobial and anticancer activities, molecular docking

## Abstract

The newly synthesized organometallic acetyl ferrocene imine ligand (HL) was obtained by the direct combination of 2-acetyl ferrocene with 2-aminothiophenol. The electronic and molecular structure of acetyl ferrocene imine ligand (HL) was refined theoretically and the chemical quantum factors were computed. Complexes of the acetyl ferrocene imine ligand with metal(II)/(III) ions (Cr(III), Mn(II), Fe(III), Co(II), Ni(II), Cu(II), Zn(II) and Cd(II)) were fabricated. They were inspected by thermal (DTG/TG), spectroscopic techniques (FT-IR, ^1^H NMR, mass, UV–Vis), molar conductivity, and CHNClM to explicate their structures. Studies using scanning electron microscope (SEM) were conducted on the free acetyl ferrocene imine ligand and its Cd(II) chelate to confirm their nano-structure. To collect an idea about the effect of metal ions on anti-pathogenic properties upon chelation, the newly synthesized acetyl ferrocene imine ligand and some of its metal chelates were tested against a variety of microorganisms, including *Bacillus subtilis*, *Staphylococcus aureus*, *Salmonella typhimurium*, *Escherichia coli*, *Aspergillus fumigatus*, and *Candida albicans*. The ligand and its metal chelate were tested for cytotoxic activity in human cancer (MCF-7 cell viability) and human melanocyte cell line HBF4. It was discovered that the Cd(II) chelate had the lowest IC_50_ of the three and thus had the prior activity. Molecular docking was utilized to investigate the interaction of acetyl ferrocene imine ligand (HL) with the receptors of the vascular endothelial growth factor receptor VEGFR (PDB ID: 1Y6a), human Topo IIA-bound G-segment DNA crystal structure (PDB ID: 2RGR), and *Escherichia coli* crystal structure (PDB ID: 3T88).

## 1. Introduction

Imine compounds are a vast class of organic compounds made by interacting primary amino compounds with aldehydes or ketones. They are frequently employed in industry and can catalyze the oxidation of organic molecules and polymers [1,2].

They are potentially capable of creating stable complexes with diverse metal ions particularly in the creation of imine metal chelates [3,4,5]. Imine ligands with sulfur and nitrogen donor atoms in their structures are effective metal chelating agents and have a wide range of therapeutic applications [6,7,8]. Imine compounds produced from 2-aminothiophenol, 2-aminophenol, 2-aminobenzoic acid, and 2-amino-3-hydroxy pyridine, in particular, have antipathogenic activity, inhibition of protein tyrosine phosphatases, and nuclease activity [9]. These activities could be owing to the inclusion of both hard and soft donor groups in a single ligand, which boosts the potential to coordinate with both hard and soft acidic metals [10].

The catalytic activity of acetyl ferrocene imine metal chelates has been investigated in a variety of processes, including oxidation, polymerization, ketones reduction, epoxidation, allylic alkylation, and Michael addition [11,12,13,14]. Acetyl ferrocene imine compounds represent an efficient class of compounds in the pharmaceutical and medicinal fields owing to their potential applications. Properties, such as antitumor, anticancer, antifungal, anticonvulsant, antibacterial, antitubercular, antimalarial, antioxidant, anti-HIV, and anti-inflammatory activities [11,15,16,17], demonstrate their importance.

Redox-active molecules with potential uses in fields such as molecular electronics materials [18,19] and physiologically active chemicals [20] have recently attracted a lot of attention. The use of ferrocenyl amines as a chelating agent for some transition metal ions has piqued the researchers’ curiosity in this study. This work aims to prepare a new Acetyl ferrocene imine derived from the condensation of 2-acetylferrocene and 2-aminothiophenol to prepare its complexes in first-row transition metal series, confirming their structure with some spectroscopic tools, elemental analyses, and thermal studies. Some theoretical studies (DFT and docking) were performed. Their biological applications as antimicrobial and anticancer agents were screened.

## 2. Experimental

### 2.1. Reagents and Materials

All of the chemical compounds utilized were analytical reagent grade (AR) and of the utmost precision. They included 2-aminothiophenol and 2-acetylferrocene, CdCl_2_, FeCl_3_.6H_2_O, CrCl_3_.6H_2_O, CoCl_2_.6H_2_O, and MnCl_2_.2H_2_O which were provided from Sigma-Aldrich while ZnCl_2_, CuCl_2_.2H_2_O, and NiCl_2_.6H_2_O were purchased from BDH, Merck, and Strem Chemicals, respectively. EtOH (90%), Et_2_O, and (CH_3_)_2_NCH were utilized as organic solvents. In most cases, de-ionized water was used in all treatments.

### 2.2. Solutions

All Stock solutions of the prepared compounds employed in the current investigation and solutions for the anticancer study were mentioned in detail in Supporting Information.

### 2.3. Instruments

All instruments employed in the current investigation were mentioned in detail in Supporting Information.

### 2.4. Molecular Structure

The Gaussian03 program suite was used to establish the acetyl ferrocene imine ligand’s molecular structure [21,22]. They were completely refined using the LANL2DZ basis set and the DFT-based B3LYP approach. The TDDFT approach (together with the LANL2DZ basic set) was used to predict the electronic transition spectra of the ligand to evaluate the influence of the solvent around the molecule. The molecular orbital contribution to HOMO and LUMO was also computed.

### 2.5. Molecular Docking

MOE 2008 software, a constrictive molecular docking software, was used to assess the different binding interactions of the most active drugs versus the receptor of Vascular endothelial growth factor receptor VEGFR (PDB ID: 1Y6a), human Topo IIA-bound G-segment DNA crystal structure (PDB ID: 2RGR), and *Escherichia coli* crystal structure (PDB ID: 3T88) [23].

Gaussian03 software was used to generate the ligand structure in PDB format. The crystal structures of the Vascular endothelial growth factor receptor VEGFR (PDB ID: 1Y6a), human Topo IIA-bound G-segment DNA (PDB ID: 2RGR), and Escherichia coli (PDB ID: 3T88) were obtained from the database of proteins (http://www.rcsb.org./pdb, accessed on 1 April 2022).

### 2.6. Synthesis of Acetyl Ferrocene Imine Ligand

Refluxing a mixture of 2-acetyl ferrocene (20.86 mmol, 4.76 g) and 2-aminothiophenol (20.86 mmol, 4.76 g) dissolved in EtOH yielded the ferrocene imine ligand (HL). After swirling the mixture under reflux for about 4 h, a dark brown solid chemical was separated. After being filtered, recrystallized, and washed with Et_2_O, it was vacuum-dried.

### 2.7. Synthesis of Acetyl Ferrocene Imine Metal Chelates

The acetyl ferrocene imine Cr(III), Mn(II), Fe(III), Co(II), Ni(II), Cu(II), Zn(II), and Cd(II) chelates were synthesized combinations of 1:1 molar mixture of hot EtOH solution (65 °C) of chloride salts (1.19 mmol) and the acetyl ferrocene imine ligand (HL) (0.4 g, 1.19 mmol). The resulting mixture was agitated for 1 h under reflux, after which the metal chelates separated. They were extracted through filtering and purified by washing with Et_2_O multiple times.

### 2.8. Biological Activity

#### 2.8.1. Anti-Pathogenic Activity

The antimicrobial activity of the compounds under investigation was screened according to the Well diffusion approach [24,25,26]. The detailed method was shown in the Supporting Information file. The biological activity values for the prepared compounds were obtained as means of duplicate experiments.

#### 2.8.2. Optimization of Anticancer Study

The cytotoxicity of the prepared compounds against the MCF-7 cell line was investigated according to employed methods in the literature [25,27]. The detailed method was shown in the Supporting Information.

## 3. Results and Discussion

### 3.1. Identification of the Acetyl Ferrocene Imine Ligand

The organometallic acetyl ferrocene imine ligand (cyclopenta-2,4-dien-1-yl(2-(1-((2-mercaptophenyl)imino)ethyl)cyclopenta-2,4-diene-1-yl)iron) (HL) which conducted by the direct combination reaction between 2-acetylferrocene and 2-amino thiophenol in hot ethanol as solvent, is a dark brown solid powder, very stable in air, and soluble in the most common organic solvents, such as methanol, ethanol, DMF, and DMSO. The elemental analyses (C, H, N, S, and M) data with the molecular formula of the free organometallic ligand, HL, are presented in Table 1. The experimental results were in good accordance with the suggested molecular formula, C_18_H_17_NSFe. The optimized structure of the free organometallic acetyl ferrocene imine (HL) ligand is represented in Figure S1. The FT-IR spectra of the organometallic acetyl ferrocene imine ligand, Table 2, show a sharp strong absorption band at 1656 cm^−1^ due to the imine group indicating the formation of the acetyl ferrocene imine ligand, while the absence of primary amine peaks at 3285 and 3267 cm^−1^ of amino thiophenol and the υ(C=O) peak at about 1700 cm^−1^ of acetyl ferrocene confirmed the ligand formation [28,29]. In addition, the IR spectrum shows a small band at 2359 cm^−1^ and a medium band at 748 cm^−1^ which was assigned to SH and CS vibrations, respectively [30]. The harmonic vibrations of the optimized structure have been analyzed theoretically. The vibrational frequencies found using quantum chemistry approaches such as DFT levels have long been known to have systematic inaccuracies. To rectify the effects of anharmonicity and the disregarded component of electron correlation, a scaling factor of 0.96 was applied for the LanL2DZ level [31,32]. Figure S2a,b represents the theoretical and experimental FT-IR spectra of HL which show characteristic bands at 2498 cm^−1^ and 1669 cm^−1^ due to υ(SH) and υ(C=N), respectively, that confirmed the experimental data. The disappearance of the NH_2_ protons in the ^1^HNMR spectrum indicated the formation of acetyl ferrocene imine ligand as there were no signals at (δ 8.34–8.96 ppm) [33,34].

**Figure 1:**
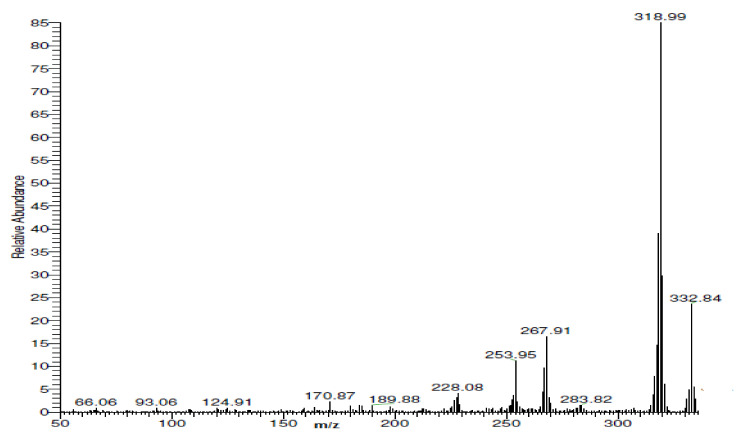
Mass spectrum of acetyl ferrocene imine ligand.

Figure 1 shows a very clear peak in the mass spectrum of the acetyl ferrocene imine ligand at *m*/*z* = 334 g/mol, with a relative intensity of 4% which is very close to the expected molecular weight of 335.24 g/mol.

#### Geometrical Optimization of the Investigated Acetyl Ferrocene Ligand

At the DFT level of theory, the full configuration of the free acetyl ferrocene imine ligand HL was accomplished [35,36]. The optimized structure’s bond distances and angles are represented in Table S1. Figure S1 shows the fully optimized shape of the free ligand as well as the atom numbering. Table 3 shows the computed quantum chemical factors. Equations (1)–(8) were used to derive further characteristics, such as E, absolute electro-negativities, chemical potentials, Pi, absolute hardness, absolute softness, global electrophilicity, global softness, S, and additional electronic charge, N_max_ [37,38,39].

∆E = E_LUMO_ − E_HOMO_ (1)

χ = −(E_HOMO_ + E_LUMO_)/2 (2)

η = (E_LUMO_ − E_HOMO_)/2 (3)

σ = 1/η(4)

P_i_ = −χ (5)

*S* = 1/2η (6)

ω = P_i_^2^/2η (7)

∆N_max_ = −Pi/η (8)

The electric and optical characteristics, as well as molecular electronic (UV–Vis) spectra and chemical reactions, are all influenced by the border molecular orbitals [40]. The energy variance, E, between the HOMO and LUMO levels reflects the ligand’s stability, and it has also aided in the development of theoretical models for describing various molecular systems’ structural and conformational barriers [41,42]. The predicted data showed that high softness indexes S and ω indicate that our HL is a soft ligand that is reactive and has flexible donation towards the cations.

The free ligand HL can accept electrons from metal ions, which decreases its energy and increases its stability; so, the electronic chemical potential is negative and the electrophilicity index is positive [43]. The high total energy negativity of the free ligand indicated its high stability.

### 3.2. Metal Chelates Characterization

#### 3.2.1. Molar Conductance and Elemental Analyses Studies

The reaction of acetyl ferrocene imine ligand with metal ions gave metal chelates which were separated in good yield (more than 70%). The experimental results of elemental analyses of the complexes were compatible with their proposed compositions and confirmed the formation of metal chelates in a ratio of 1:1 (Metal: ligand) (Table 1). Some metal chelates showed high stability as they have a high melting point (>300 °C), other complexes had melting points ranging from 125 °C to 260 °C. The percent yield of the complexes is in the range of 70–90%. All metal chelates are dissolved in most organic solvents such as DMSO and DMF but insoluble in ethanol, methanol, and acetone. Molar conductivity of metal chelates showed that Cr(III), Mn(II) and Fe(III), Co(II), Ni(II), and Cu(II) chelates were electrolytes but Zn(II) and Cd(II) chelates were non-electrolytes (Table 1).

#### 3.2.2. Vibration Spectra for the Prepared Compounds

The IR spectral data of acetyl ferrocene imine ligand HL and its metal chelates and their possible assignments are given in Table 2. The IR spectra of the metal chelates showed a broad peak in the range 3401–3443 cm^−1^ which demonstrated that υ(OH) of coordinated water. Three distinct bands were seen in the free acetyl ferrocene imine ligand at 2359 cm^−1^, 1656 cm^−1^, and 748 cm^−1^ due to υ(SH) [44], υ(C=N) [45,46], and υ(CS) [44] vibrations, respectively. In the metal chelates, these bands altered to be in ranges of 2337–2376 cm^−1^ for υ(SH), 1607–1644 cm^−1^ for υ(C=N), and 748–755 cm^−1^ for υ(CS) which differ also in intensity. This shift indicated coordination with metal ions in the sites of nitrogen of the imine group and sulfur of thiophenol which resulted in the appearance of new bands M-S, M-N, and M-O (of coordinated water) in ranges of 450–495 cm^−1^ [47], 476–580 cm^−1^, and 562–624 cm^−1^, respectively [48]. Also coordinated water showed bands at 820–878 cm^−1^ and 881–1060 cm^−1^ [49]. Based on these findings, the HL acted as a neutral bidentate ligand that coupled to metal chelates via NS sites.

#### 3.2.3. HNMR Spectroscopic Studies

The ^1^H NMR data for the free ligand and Zn(II) complex were recorded in DMSO-d_6_ and showed four significant signals at (δ 1.05 ppm) [49], (δ 3.32 ppm) [30], (δ 4.23–4.77 ppm) [50], and (δ 5.42–7.06 ppm) [51] due to 3H of the methyl group of azomethine, SH group, 9H of acetyl ferrocene, and 4H of an aromatic moiety, respectively. The spectrum of Zn(II) complexes showed some variations from the ligand which explained the formation of new bonds with metal ions due to the shift of the signals of the azomethine methyl group, SH, 2-acetylferrocene, and aromatic moiety to (δ 1.25 ppm), (δ 3.48 ppm), (δ 4.18–4.96 ppm), and (δ 6.42–7.96 ppm), respectively.

#### 3.2.4. UV–Vis Absorption Studies

**Figure 2:**
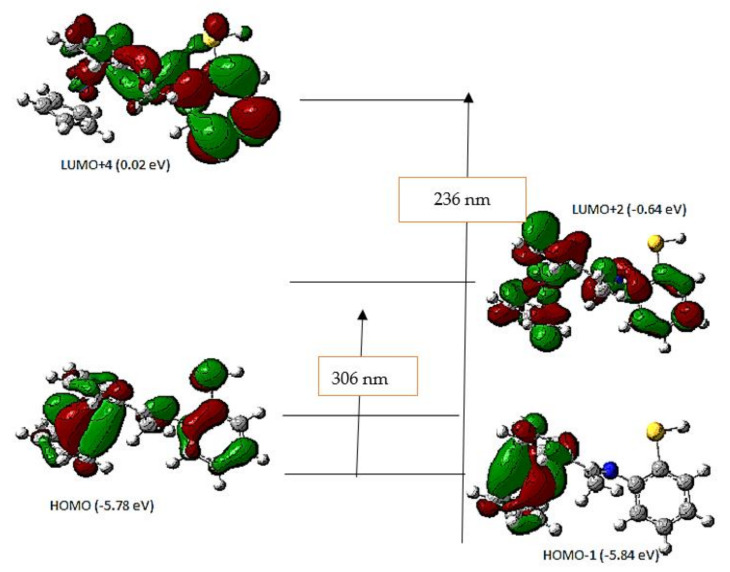
Possible molecular orbitals’ transitions of the acetyl ferrocene imine ligand.

Molecular transition spectra of the free acetyl ferrocene imine ligand and its metal chelates were recorded in (ethanol: DMF) solution in equal ratios. The absorption spectra for the free ligand (HL) exhibit two main peaks, the first appears at 236 nm which was ascribed to benzene and ferrocene π-π* transitions and the second peak appears at 306 nm which was assigned to the azomethine n-π* transitions [52]. These bands moved in metal chelates to 233 nm for π-π* sulfur atom transitions and 287–307 nm for n-π* transitions. In addition, there is the appearance of new bands at 537–630 nm which indicated d-d splitting. Theoretically, the two main absorption bands at 236 and 325 nm (in DMF) are in agreement with the experimental spectrum (Figure S3a,b). The transitions of HOMO-1 to LUMO + 4 with excitation energy of 5.86 eV give rise to the higher energy band at 236 nm. The lower energy band, which occurred at 325 nm and showed a transformation from HOMO to LUMO + 2 with excitation energy of 5.14 eV, is depicted in Table 4, and Figure 2 illustrates the molecular orbital transitions.

#### 3.2.5. Thermal Analysis

Thermal degradations were performed under an air environment at a rate of heating of 10 °C min^−1^ from ambient temperature to 1000 °C. Table 5 and Figure S4 summarize the degradable segments against different temperature ranges. The free acetyl ferrocene imine ligand was decayed in two stages at T_s_ = 272 and 317 °C with a total loss of mass of 47.86% (predicted = 47.39%) and the remaining contaminated FeO residue. The Cr(III) complex demonstrated two degradation stages. The first occurred in the temperature range 40–500 °C, with a found loss of mass = 38.05% (predicted = 38.27%) due to loss of two water molecules, three HCl molecules, and a benzene molecule. The second degradation stage occurred at T_s_ = 882 °C with a found loss of mass = 16.31% (predicted = 16.09%) due to loss of C_2_H_16_NSO_0_._5_ moiety leaving ten carbon atoms contaminating metals oxides as residues.

The Mn(II) complex decayed through four stages; firstly, removal of water of crystallization at T_s_ = 56 °C; next, two degradation stages occurred at 100–245 °C in which 2H_2_O, HCl, and NH_3_ molecules were lost with the found loss of mass of 15.31% (predicted = 15.72%). Finally, the last degradation stage occurred at temperature maxima of 252 °C due to the loss of C_6_H_17_SCl moiety of the ligand leaving MnO, FeO, and 12C as residues with a total found loss of mass of 50.07% (predicted = 49.52%). For [Fe(HL)(H_2_O)_3_Cl] Cl_2_.3H_2_O, it showed three degradation stages; the 1st stage occurred at T_s_ = 67 °C indicating a loss of three molecules of water of crystallization. The 2nd and 3rd degradation stages took place in the range of 130–1000 °C at which C_7_H_23_Cl_3_NSO_0_._5_ molecules were lost with the found loss of mass of 44.99% (predicted = 44.11%), leaving contaminated iron oxides. The Co(II) complex TG curve showed three degradation stages where the first stage occurred in a temperature range of 10–100 °C with a found loss of mass of 4.27% (predicted = 3.25%) due to loss of hydrated water molecule. The last two stages occurred in the range of 100–1000 °C at which two molecules of coordinated water and the organic moiety of the ligand were lost leaving cobalt and iron oxides residues contaminated with carbon with a total loss of mass (found = 46.72%, predicted = 45.47%). In the temperature range 45–125 °C, the TG curve of the [Ni(HL)(H_2_O)_3_Cl] Cl.2H_2_O complex demonstrated a peak at 81 °C, equivalent to a weight loss of 7.52% (found weight loss = 6.48%) related to the removal of two hydrated water molecules. The loss of the water molecule and C_6_H_21_NSCl_2_ fragment may be attributed to the second and third phases of thermal breakdown, which occurred in the range 125–1000 ºC with two maxima at 369 and 876°C (found 41.17%; predicted 41.04%). At the end of the TG curve, NiO and FeO have remained as residues with carbon contamination. The Cu(II) complex produced a three-stage breakdown pattern from 30 °C to 1000 °C. The first stage was a single-stage between 30 and 120 °C, with a high temperature of 92 °C, signifying the loss of hydrated water molecules with a loss of mass of 4.05% (predicted = 3.32%). Within the temperature range of 120–240 °C and with a temperature maximum of 140 °C, the second stage was similar to the first stage, representing the loss of H_2_O and HCl molecules with a loss of mass of 9.38% (predicted = 10.06%). The final stage was to reflect the loss of the C_4_H_20_NSCl fragment with a loss of mass of 26.64% (predicted = 27.58%) between 240 and 1000 °C. At the end of the thermogram, contaminated Cu(II) and Fe(II) oxides were residues. On the other hand, The [Zn(HL)Cl_2_].2H_2_O complex describes four stages of breakdown. The complex loses H_2_O (hydrated) and ammonia gas with a found loss of mass of 11.30% (predicted = 10.54%) in the first stage in the temperature range of 20–170 °C, with one maximum at 92 °C. The second stage occurred at 170–360 °C with T_s_ = 318 °C, in which the complex loses the C_5_H_5_ molecule with a found loss of mass of 12.71% (predicted = 12.81%). The final two stages at 360–1000 °C correspond to the breakdown of C_9_H_12_SCl with a loss of mass of 36.29% (predicted = 36.95%). Carbon-contaminated ZnO and FeO were left as the degradation residue. The overall weight loss amounted to 60.26% (predicted = 60.30%). From 95 to 1000 °C, the Cd(II) complex disintegrated in four phases as follows. The first two phases were carried out at 95–530°C, resulting in the loss of NH_3_, CH_4_, C_5_H_5_, and 2HCl molecules, with a loss of mass of 30.76% (predicted = 30.84%). The final stages described the breakdown of the C_12_H_7_S molecule with a loss of mass of 32.65% (predicted = 33.00%) with T_s_ = 569 and 677 °C. Finally, FeO and CdO were the residues with a total found loss of mass of 63.51% (predicted = 63.84%). The presence of carbon contamination with the residues of the ligand and all complexes except the Cd(II) complex can be explained by the high stability of these compounds as they cannot be decomposed completely up to 1000 °C.

Carbon residues were found in practically all metal chelates with metal oxides, which might be explained by the high stability of the complexes including ferrocene derivatives. Complexation with metals strengthened their stability, thus, they could not be totally dissolved at high temperatures.

#### 3.2.6. SEM Inspection

**Figure 3:**
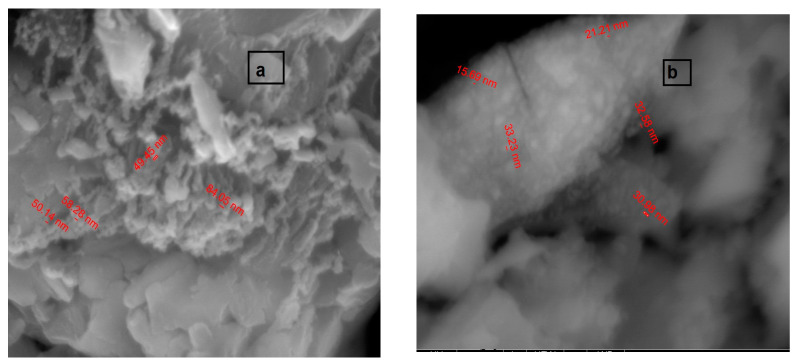
SEM graphs of (**a**) ligand and (**b**) [Cd(HL)(H_2_O)_2_Cl_2_] complex.

Scanning electron microscope (SEM) images of the ligand and Cd(II) complex at 50,000× magnifications indicated the existence of these compounds at nanoscale (Figure 3a,b). The free ligand appeared as a coral reef structure in size 49.45 nm, while upon complexation with Cd(II), it converted to a spongy shape with size 15.69 nm. These data confirmed that the surface morphology of the complex differs from that of the ligand and this can account for the binding of ligand to Cd(II) ion as it causes changes in the skeleton of the ligand. The presence of the ligand and Cd(II) complex in nanosize can be assigned to the improved biological and anticancer activities.

#### 3.2.7. Structural Manipulation

**Figure 4:**
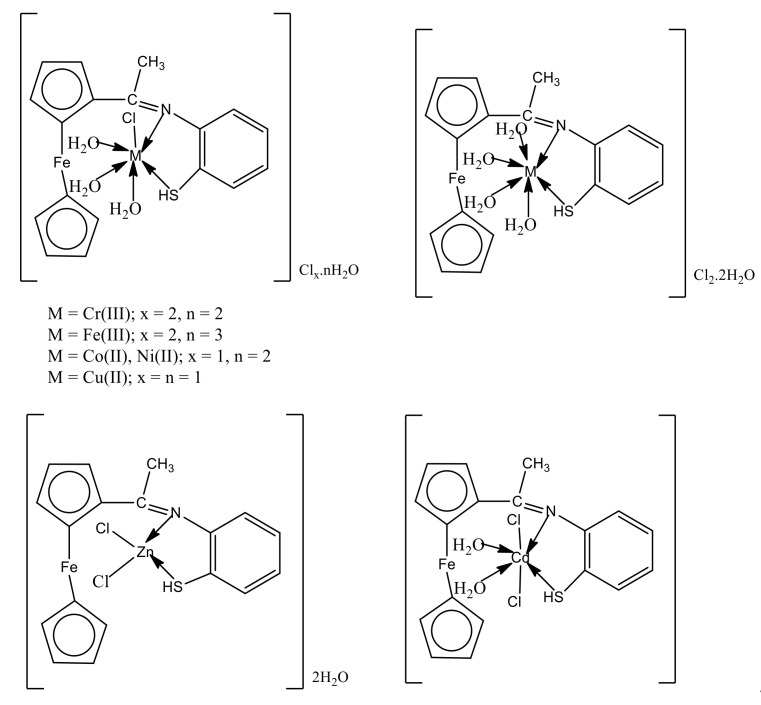
Structure of metal complexes of Schiff base ligand.

Elemental studies, IR, ^1^H NMR, mass and UV–visible spectra, molar conductivity, SEM, and thermal investigations (DTG/TG), were used to confirm the structures of the free organometallic acetyl ferrocene imine ligand (HL) and its metal chelates. As illustrated in Figure 4, the suggested structural formulas of the investigated metal chelates were described as three types of coordination (8), and their general formulas were [M(HL)(H_2_O)_3_Cl]Cl_x_.nH_2_O (M = Cr(III); x = 2, n = 2), (M = Fe(III); x = 2, n = 3), (M = Co(II) and Ni(II); x = 1, n = 2) and (M = Cu(II), x = n = 1), [M(HL)(H_2_O)_x_Cl_2_].nH_2_O (M = Zn(II); x = 0, n = 2), (M = Cd(II); x = 2, n = 0) and [Mn(HL)(H_2_O)_4_]Cl_2_.2H_2_O.

### 3.3. Antimicrobial Efficiency

**Figure 5:**
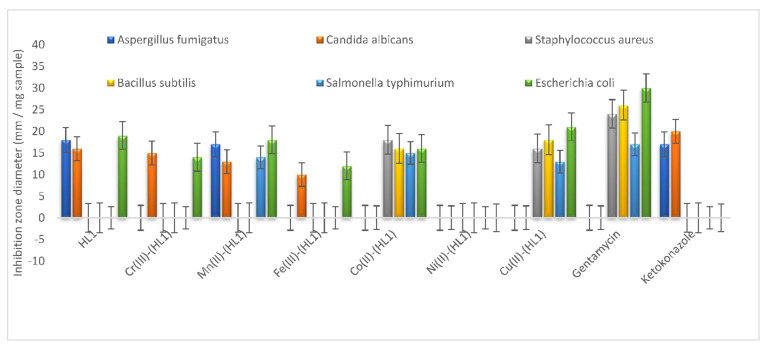
Biological efficiency of HL and its metal chelates.

The newly synthesized acetyl ferrocene imine ligand, Cr(III), Mn(II), Fe(III), Co(II), Ni(II), and Cu(II) chelates were screened for their antifungal and antibacterial activities against **(***Aspergillusfumigatus* and *Candidaalbicans*) and (*Staphylococcusaureus* and *Bacillussubtilis*, gram-positive, *Salmonellatyphimurium* and *Escherichia coli*, gram negative), respectively. The results are represented in Table S2 and Figure 5. The free ligand showed the highest antifungal activity (18 and 16 mm/mg), followed by Mn(II) complex which had inhibition zone diameter values of 17 and 13 mm. Co(II) and Cu(II) complexes showed high antibacterial activities, and the values can be arranged as follows:

- For *Staphylococcus aureus* Co(II) > Cu(II) but for *Bacillus subtilis* Cu(II) > Co(II).
- For *Salmonella typhimurium* Co(II) > Cu(II) but for *Escherichia coli* Cu(II) > Co(II).

It was found that the free ligand had no antibacterial activities against *Staphylococcus aureus, Bacillus subtilis,* and *Salmonella typhimurium*, while some complexes had activities. In addition, it is observed that free ligand had activity against *E. coli* is lower than some complexes, this can be explained.

The enhancement in the antibacterial activity of the investigated acetyl ferrocene metal chelates compared to acetyl ferrocene imine ligand can be supported by Overtone’s concepts [53,54,55]. Because chelation strengthens the ligand’s ability to operate as more effective and potent bactericidal agents by limiting bacterial growth, metal complexes were discovered to have higher zones of inhibition than free ligands. Because of the partial sharing of positive charge on metal with the hetero donor atoms of the ligand and also due to electron delocalization over the entire chelate ring system, chelation diminishes the polarity of the metal ion significantly. Lipids and polysaccharides are essential components of the cell wall and membranes, and they are excellent for metal–ion interaction. Apart from that, the cell walls contain phosphates, carbonyl, and cystenyl ligands that help maintain the membrane’s integrity by acting as a diffusion barrier and also provide adequate support [56].

The differences in metal complex action against different bacteria are due to differences in the ribosomes of the microbial cells or the impermeability of the microbes’ cells. The reduced activity of complexes relative to others could be due to limited lipid solubility, preventing the metal ion from reaching the cell wall’s favorable site of action and interfering with normal cell activity. Although chelation plays an important role in evaluating antibacterial activity of complexes, other variables, such as solubility, size, dipole moment, coordinating sites, redox potential of metal ions, solubility, bond length between metal and ligand, geometry of complexes, steric, pharmacokinetic, concentration, and hydrophobicity also play a role. As a result of the findings, it is clear that increased antibacterial activity of metal complexes may be attributable to a complex combination of various additional mechanisms in addition to chelation [57].

According to the equation, activity index (A) = ((Inhibition zone of compound (mm)/Inhibition zone of the standard drug (mm)) × 100) [57,58], the activity indexes of the tested substances were computed and plotted in Figure S5a,b. The figures show that the free ligand and Mn(II) complex had high activity indexes, while Ni(II) complex had no activity index.

### 3.4. Anticancer Activities

**Figure 6:**
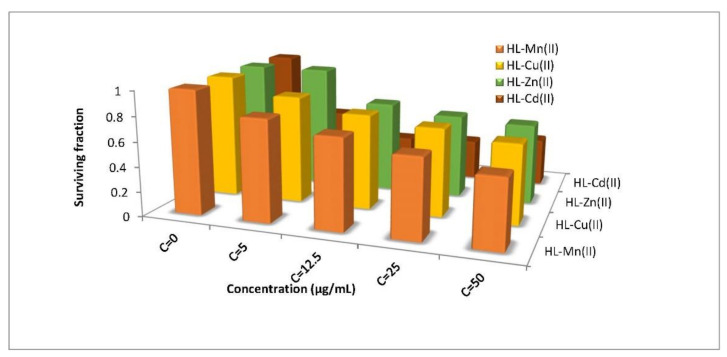
Anticancer activity of the free acetyl ferrocene imine ligand and some metal chelates.

The efficiency of the free organometallic acetyl ferrocene imine ligand and its metal chelates as a potential antitumor drug has been tested in vitro on the MCF7 cell line (derived from human breast cancer) and HBF4 cell line (derived from normal human melanocyte). The results for MCF7 expressed as the concentration of the complex required to inhibit the tumor cell growth by 50% (IC_50_) are recorded in Table S3 and these data are represented in Figure 6. Only four complexes were found to have a certain inhibitory effect of >70%, while the free ligand and other complexes could not inhibit >70% of cancerous cells. Mn(II), Cu(II), Zn(II), and Cd(II) complexes have MCF7 inhibitory effect while the most potent with the lowest IC_50_ value (5.57 µg/mL) was Cd(II) which showed the most potent cytotoxic results versus the cell line which may be revealed to its nano-size and morphology. In addition, Co(II), Cu(II), Zn(II), and Cd(II) chelates demonstrate surviving fractions 52–62% when tested for HBF4 cell line at concentration 100 µg/mL. By comparing IC_50_ of the Cd(II) complex with that of 3-(butylamino)-2-[(*E*)-2-(2-methoxyphenyl) ethenyl]-4*H*-chromen-4-one compound [59], we found that Cd(II) (5.57 µg/mL) was three times lower than the other compound (15.62 µg/mL) which had high cytotoxic effect. This provides motivation to continue studies on our complex to develop a new antibreastic cancer drug.

### 3.5. Molecular Docking

For the purpose of rational drug development and discovery, molecular docking is a vital tool for predicting the most preferred mode of binding of a small molecule to its targeted protein, between the targeted molecule (receptor) and the desired compounds (such as ligand and its metal chelates) with low binding free energy but high binding affinity. The MOE 2008 program suite was used to dock the free organometallic ligand HL and the Cd(II) chelate with the vascular endothelial growth factor receptor VEGFR (PDB ID: 1Y6a), the crystal structure of human Topo IIA-bound G-segment DNA (PDB ID: 2RGR), and crystal structure of *Escherichia coli* (3T88).

Antibodies (inhibitors) to the vascular endothelial growth factor receptor (VEGFR) are important in the treatment of a variety of malignancies [60]. As a result, VEGFR-2 has been chosen as the biological target for docking investigations on active molecules. Table 6 shows the binding energies of the free ligand HL and Table 7 shows the binding energies of Cd(II) complex. From these data, it was found that the Cd(II) complex with binding energy −14.3 kcal/mol is more favorable than the free ligand (−2.6 kcal/mol). It is also more potent than the 2-(4-Benzothiazol-2-yl-phenylimino)-5-(4-nitro-benzylidine)-thiazolidin-4-one compound which had a binding energy of −13.3 kcal/mol [60].

This research was also expanded to look at the mechanism of action of our drugs with the human DNA topoisomerase II enzyme. For medicinal chemists working on anticancer medicines, DNA topoisomerases were a key molecular target. The free organometallic ligand bonded to the enzyme via hydrogen, ionic, and metallic bonds with higher positive binding energy (−1.9 kcal/mol) than the Cd(II) complex which exhibits a binding energy of −20.7 kcal/mol [61,62].

**Figure 7:**
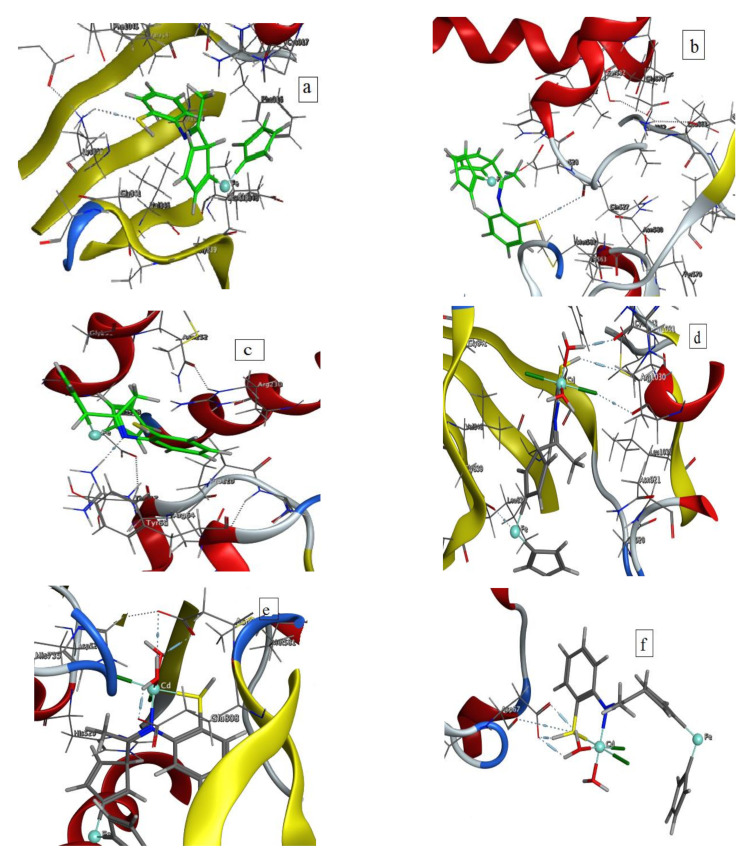
3D plot of the interaction between the free ligand with receptors of (**a**) 1Y6a, (**b**) 2rgr and (**c**) 3 T88 and 3D plot of the interaction between Cd(II) chelate with receptors of (**d**) 1Y6a, (**e**) 2rgr, and (**f**) 3 T88.

On studying binding of HL and its Cd(II) chelate with the crystal structure of *E.coli*, it was found that both compounds were bounded by H-bonds with *E.coli* receptor and upon complexation, the binding energy became more negative. Figure 7 shows 3D interactions of both the free ligand and Cd(II) chelate.

From the previous data, it was concluded that the Cd(II) complex had the lowest binding energy with 2RGR receptor (−20.7 kcal/mol) which indicated that human DNA topoisomerase IIα enzyme was the most effective binding protein.

## 4. Conclusions

The metal chelates of Cr(III), Mn(II), Fe(III), Cu(II), Ni(II), Co(II), Zn(II), and Cd(II) were synthesized from the bi-dentate ligand derived from condensation of 2-acetyl ferrocene with 2-aminothiophenol (HL) and characterized using different spectroscopic methods. The ligand acted as neutral bidentate (NS) ligand, and all complexes showed octahedral geometry except Zn(II) was tetrahedral. All complexes are electrolytes, except Zn(II) and Cd(II) complexes, which are non-electrolytes, according to molar conductivity data. The complexes have M(HL)-type compositions with generic formulae, according to elemental analysis data, [M(HL)(H_2_O)_3_Cl] Cl_z_.nH_2_O (M = Cr(III); x = 2, n = 2), (M = Fe(III); x = 2, n = 3), (M = Co(II) and Ni(II); x = 1, n = 2) and (M = Cu(II), x = n = 1), [M(HL)(H_2_O)_x_Cl_2_].nH_2_O (M = Zn(II); x = 0, n = 2), (M = Cd(II); x = 2, n = 0) and [Mn(HL)(H_2_O)_4_]Cl_2_.2H_2_O. SEM graphs showed our ligand and the Cd(II) complex in nanosize with different shapes. The antimicrobial test referred that the HL had the highest antifungal activity while Co(II) and Cu(II) complexes had high antibacterial activities. Furthermore, cytotoxicity of the Cd(II) complex revealed that it has better anticancer activity than the others, with an IC_50_ of 5.57 g/mL, suggesting that it could be used in clinical trials or as a biological agent.

## Figures and Tables

**Table 1:** Analytical and physical data of organometallic acetyl ferrocene imine ligand and its metal chelates.

Compound (Molecular Formula)	Colour (%yield)	M.p. (°C)		% Found (Calcd.)					Λ_m_ Ω^−1^mol^−1^ cm^2^
			C	H	N	S	Cl	M	
HL (C_18_H_17_NSFe)	Dark brown (85)	190	64.38 (64.40)	5.07 (4.93)	4.17 (4.20)	9.54 (9.95)	------	16.84 (16.50)	------
[Cr(HL)(H_2_O)_3_Cl]Cl_2_.2H_2_O (C_18_H_27_NO_5_SCl_3_CrFe)	Dark brown (86)	>300	36.99 (37.00)	4.62 (5.00)	2.40 (2.78)	5.50 (5.55)	18.54 (18.93)	18.58 (19.00)	138
[Mn(HL)(H_2_O)_4_]Cl_2_.2H_2_O (C_18_H_29_NO_6_SCl_2_MnFe)	Brown (84)	258–260	36.70 (36.73)	4.93 (5.03)	2.38 (2.82)	5.44 (5.65)	12.06 (12.56)	18.35 (18.55)	111
[Fe(HL)(H_2_O)_3_Cl]Cl_2_.3H_2_O (C_18_H_29_NSO_6_Cl_3_Fe_2_)	Brown (85)	>300	35.31 (35.45)	4.78 (5.00)	2.31 (2.30)	5.28 (5.68)	17.16 (17.20)	18.63 (18.96)	100
[Co(HL)(H_2_O)_3_Cl]Cl.2H_2_O (C_18_H_27_NO_5_SCl_2_CoFe)	Brown (70)	>300	38.95 (39.50)	4.87 (5.30)	2.52 (2.70)	5.77 (5.80)	12.40 (12.50)	20.65 (20.80)	78
[Ni(HL)(H_2_O)_3_Cl]Cl.2H_2_O (C_18_H_27_NSO_5_Cl_2_NiFe)	Dark brown (80)	237–240	38.88 (39.37)	4.86 (5.30)	2.52 (2.60)	5.76 (5.71)	12.38 (12.55)	20.70 (20.78)	82
[Cu(HL)(H_2_O)_3_Cl]Cl.H_2_O (C_18_H_25_NSO_4_Cl_2_CuFe)	Dark brown (90)	197–200	39.85 (40.00)	4.41 (4.50)	2.58 (2.90)	5.90 (6.00)	13.10 (13.70)	22.14 (22.50)	54
[Zn(HL)Cl_2_].2H_2_O (C_18_H_21_NSO_2_Cl_2_ZnFe)	Dark brown (75)	125–128	42.56 (42.23)	4.24 (4.60)	2.76 (2.42)	6.31 (6.50)	13.99 (14.00)	23.94 (24.28)	18
[Cd(HL)(H_2_O)_2_Cl_2_] (C_18_H_21_NSO_2_Cl_2_CdFe)	Dark brown (72)	157–160	38.05 (38.18)	3.39 (3.50)	2.52 (2.48)	5.77 (6.00)	12.10 (12.30)	30.39 (30.60)	4

**Table 2:** IR spectra (4000–400 cm^−1^) of HL ligand and its metal chelates.

HL.	HLCr	HLMn	HLFe	HLCo	HLNi	HLCu	HLZn	HLCd	Assignment
-------	3408 br	3426 br	3401 br	3430 br	3410 br	3430 br	3437 br	3443 br	υ(OH)
2359 s	2348 s	2337 s	2376 s	2357 m	2369 w	2372 w	2367 w	2355 s	υ(SH)
1656 sh	1626 m	1628 m	1641 w	1644 sh	1634 m	1642 m	1616 m	1607 sh	υ(C=N)
-------	878 w, 1060 w	876 w, 970 w	843 s, 920 s	864 m, 990 s	825 w, 980 s	820 m, 883 w	826 m, 893 w	823 w, 881 w	υ(H_2_O)
748 m	751 w	750 s	748 w	752 w	751 w	752 w	755 m	748 sh	υ(C-S)
-------	608 w	562 w	602 w	613 w	615 s	617 w	624 w	614 sh	M-O stretch of coordinated water
-------	550 s	476 s	520 s	550 s	537 s	570 s	580 s	560 s	M-N
-------	450 s	461 s	489 s	470 m	450 s	468 w	491 w	495 w	M-S

**Table 3:** The different quantum chemical parameters of the free acetyl ferrocene imine ligand.

**The Calculated Quantum Chemical Parameters**	
E (a.u.)	−884.28
Dipole moment (debey)	2.1158
E_HOMO_ (eV)	−5.78
E_LUMO_ (eV)	−1.58
ΔE (eV)	4.20
χ (eV)	3.68
η (eV)	2.1
σ (eV)^−1^	0.48
P_i_ (eV)	−3.68
S (eV)^−1^	0.24
ω (eV)	3.22
ΔN_max_	1.75

**Table 4:** Main UV-calculated optical transitions with composite ion in terms of molecular orbitals of free ligand.

**Compound**	**Transition**	**Excitation Energy (eV)**	**λ_max_ Calcd. nm/(eV)**	**λ_max_ Exp. nm/(eV)**
**HL**	HOMO→LUMO + 2 (48.9%)	5.14	325 (3.81)	306 (4.06)
	HOMO-1→LUMO + 4 (66.1%)	5.86	236 (5.24)	236 (5.26)

**Table 5:** Thermoanalytical results (DTG/TG) of the organometallic acetyl ferrocene imine ligand and its metal chelates.

**Complex**	**TG Range** **(°C)**	**DTG_max_ (°C)**	n *	**Mass Disposal Total Mass Disposal** **Found (Calcd)%**	**Assignment**	**Residues**
HL	90–1000	272, 317	2	46.59 (47.39) 47.86 (47.39)	- Removal of C_8_H_17_NS.	Fe + 10C
HLCr	40–500	71, 250	1	38.05 (38.27)	- Removal of 3HCl, C6H6, and 2H2O. - Removal of C_2_H_16_NSO_0_._5_.	½Cr_2_O_3_ + FeO + 10C
				16.31 (16.09)		
	500–1000	882	1	54.88 (54.02)		
HLMn	15–100 100–245 245–1000	56 109, 160 252	1 2 1	7.01 (6.32) 15.31 (15.72) 27.48 (27.48) 50.07 (49.52)	- Removal of 2H_2_O. - Removal of 2H_2_O, HCl and NH_3_ - Removal of C_6_H_17_SCl.	MnO + FeO + 12C
HLFe	45–130 130–1000	67 179, 606	1 2	7.74 (8.90) 44.99 (44.11) 52.98 (51.57)	- Removal of 3H_2_O. - Removal of C_7_H_23_Cl_3_NSO_0_._5_.	½Fe_2_O_3_ + FeO + 11C
HLCo	10–100 100–1000	85 125, 269	1 2	4.27 (3.25) 41.41 (42.20) 46.72 (45.47)	- Removal of H_2_O. - Removal of 2H_2_O and C_5_H_21_NSCl_2_.	CoO + FeO + 13C
HLNi	45–125 125–1000	81 369, 876	1 2	7.52 (6.48) 41.17 (41.04) 51.12 (52.48)	- Removal of 2H_2_O. - Removal of H_2_O and C_6_H_21_NSCl_2_.	NiO + FeO + 12C
HLCu	30–120 120–240 240–1000	92 140 410	1 1 1	4.05 (3.32) 9.38 (10.06) 26.64 (27.58) 40.06 (40.96)	- Removal of H_2_O. - Removal of H_2_O and HCl. - Removal of C_4_H_20_NSCl.	CuO + FeO + 14C
HLZn	20–170 170–360 360–1000	92 318 558, 931	1 1 2	11.30 (10.54) 12.71 (12.81) 36.29 (36.95) 60.26 (60.30)	- Removal of H_2_O and NH_3_. - Removal of C_5_H_5_. - Removal of C_9_H_12_SCl.	ZnO + FeO + 4C
HLCd	95–530 530–1000	196, 259 569, 677	2 2	30.76 (30.84) 32.65 (33.00) 63.51 (63.84)	- Removal of NH_3_, CH_4_ and 2HCl. - Removal of C_5_H_5_. - Removal of C_12_H_7_S.	CdO + FeO

n * = number of decomposition steps.

**Table 6:** Energy values obtained in docking calculations of HL with crystal structure of (1Y6a), the receptors of (2RGR), crystal structure of *Escherichia coli* (3T88).

**Receptor**	**Ligand Moiety**	**Receptor Site**	**Interaction**	**Distance (°A)**	**E (kcal/mol)**
1Y6a	C16	O GLU 915 (A)	H-donor	3.33	−0.7
	O4	ND2 ASN 921 (A)	H-acceptor	3.04	−2.6
	5-ring	CD1 LEU 838 (A)	pi-H	4.07	−0.8
	6-ring	CG1 VAL 846 (A)	pi-H	3.83	−0.8
	S35	NZ LYS 866 (A)	H-acceptor	3.61	−1.3
	6-ring	CG2 VAL 846 (A)	pi-H	4.14	−0.8
2RGR	S35	O GLN 527 (A)	H-donor	10.14	−0.8
	Fe9	OD2 ASP 528 (A)	metal	7.11	−1.9
	C15	MG MG 1 (A)	ionic	8.28	−1.6
	6-ring	CG LYS 603 (A)	pi-H	6.87	−0.9
	6-ring	CE LYS 603 (A)	pi-H	8.13	−1.2
3T88	N20	NH1 ARG 230 (A)	H-acceptor	3.06	−1.1
	5-ring	CB ASP 67 (A)	pi-H	4.22	−0.8

**Table 7:** Energy values obtained in docking calculations of Cd(II) chelate with crystal structure of (1Y6a), the receptors of (2RGR), crystal structure of *Escherichia coli* (3T88).

**Receptor**	**Ligand Moiety**	**Receptor Site**	**Interaction**	**Distance (°A)**	**E (kcal/mol)**
**1Y6a**	S35	OD1 ASN 1031 (A)	H-donor	3.53	−1.6
	O43	O ARG 1030 (A)	H-donor	2.74	−1.2
	O43	OD1 ASN 1031 (A)	H-donor	2.89	−14.3
	Fe9	O LEU 838 (A)	metal	2.28	−2.8
**2RGR**	O43	OE2 GLU 808 (A)	H-donor	2.81	−20.7
	O43	OD1 ASP 809 (A)	H-donor	2.79	−3.8
	O43	OD2 ASP 809 (A)	H-donor	3.54	−0.9
	N20	OE2 GLU 808 (A)	ionic	3.22	−3.2
	S35	OD1 ASP 809 (A)	ionic	3.99	−0.5
	O40	OD1 ASP 809 (A)	ionic	3.51	−1.9
	O43	OE2 GLU 808 (A)	ionic	2.81	−5.9
	O43	OD1 ASP 809 (A)	ionic	2.79	−6
	O43	OD2 ASP 809 (A)	ionic	3.54	−1.7
**3T88**	S35	O ASP 67 (A)	H-donor	3.65	−0.7
	S35	OD2 ASP 67 (A)	H-donor	3.11	−5.2
	S35	OD1 ASN 68 (A)	H-donor	3.14	−2.3
	O40	OD1 ASN 68 (A)	H-donor	2.87	−10.1
	O43	OD1 ASP 67 (A)	H-donor	2.96	−13.4
	O43	OD2 ASP 67 (A)	H-donor	2.61	−7.1
	N20	OD1 ASP 67 (A)	ionic	3.72	−1.2
	S35	OD2 ASP 67 (A)	ionic	3.11	−3.8
	O43	OD1 ASP 67 (A)	ionic	2.96	−4.8
	O43	OD2 ASP 67 (A)	ionic	2.61	−7.6

## Data Availability

The raw/processed data generated in this work are available upon request from the corresponding author.

## Supplementary Materials

The following supporting information can be downloaded at: https://www.mdpi.com/article/10.3390/ma15103678/s1, Table S1. The different optimized parameters of the free Schiff base ligand; Table S2. Biological activity of organometallic Schiff base (HL) and its metal complexes; Table S3. Anticancer activity of Schiff base ligand and its metal complexes; Figure S1. The optimized structure of the newly synthesized acetyl ferrocene imine ligand; Figure S2. IR spectra of the free ligand (a) theoretical and (b) experimental; Figure S3. UV-Vis spectra of HL (a) theoretical and (b) experimental; Figure S4. Thermograms of (a) HL, (b) HL-Cr(III), (c) HL-Mn(II), (d) HL-Fe(III), (e) HL-Co(II), (f) HL-Ni(II), (g) HL-Cu(II), (h) HL-Zn(II) and (i) HL-Cd(II) complexes; Figure S5. Activity indexes of HL and its metal complexes against tested microorganisms.

## References

[1] Arulmurugan S., Kavitha P.H., Venkatraman R.P. (2010). Biological activities of Acetyl ferrocene imine and its complexes: A review. Rasayan J. Chem..

[2] Al-Saeedi S.I., Abdel-Rahman L.H., Abu-Dief A.M., Abdel-Fatah S.M., Alotaibi T.M., Alsalme A.M., Nafady A. (2018). Catalytic Oxidation of Benzyl Alcohol Using Nanosized Cu/Ni xzSchiff-Base Complexes and Their Metal Oxide Nanoparticles. Catalysts.

[3] Abd El-Lateef H.M., Khalaf M.M., Shehata M.R., Abudief A.M. (2022). Fabrication, DFT Calculation, and Molecular Docking of Two Fe (III) Imine Chelates as Anti-COVID-19 and Pharmaceutical Drug Candidate. Int. J. Mol. Sci..

[4] Abu-Dief A.M., El-Khatib R.M., Aljohani F.S., Al-Abdulkarim H.A., Alzahrani S., El-Sarrag G., Ismael M. (2022). Synthesis, structuralelucidation, DFT calculation, biological studies and DNA inter-action of some aryl hydrazone Cr^3+^, Fe^3+^, and Cu^2+^ chelates. Comput. Biol. Chem..

[5] Shafaatian B., Mousavi S.S., Afshari S. (2016). Synthesis, characterization, spectroscopic and theoretical studies of new zinc(II), copper(II) and nickel(II) complexes based on imine ligand containing 2-aminothiophenol moiety. J. Mol. Struct..

[6] Mishra A.K., Manav N., Kaushik N.K. (2005). Organotin(IV) complexes of thiohydrazones: Synthesis, characterization and antifungal study. Spectrochim. Acta A.

[7] Aljohani E.T., Shehata M.R., Abu-Dief A.M. (2021). Design, synthesis, structural inspection of Pd^2+^, VO^2+^, Mn^2+^, and Zn^2+^ chelates incorporating ferrocenyl thiophenol ligand: DNA interaction and pharmaceutical studies. Appl. Organomet. Chem..

[8] Abu-Dief A.M., El-Khatib R.M., Salah M.E., Alzahrani S., Alkhatib F., El-Sarrag G., Ismael M. (2021). Synthesis and intensive characterization for novel Zn (II), Pd (II), Cr (III) and VO (II)-Schiff base complexes; DNA-interaction, DFT, drug-likeness and molecular docking studies. J. Mol. Struct..

[9] Kavita P., Reddy K.L. (2014). Synthesis, Structural Characterization, and Biological Activity Studies of Ni(II) and Zn(II) Complexes. Bioinorg. Chem. Appl..

[10] Soliman A.A., Linert W. (2007). Structural features of ONS-donor salicylidene Acetyl ferrocene imine complexes. Mon. Chem..

[11] Rudbari H.A., Iravani M.R., Moaza V., Askari B., Khorshidifard M., Habibi N., Bruno G. (2016). Synthesis, characterization, X-ray crystal structures and antibacterial activities of Acetyl ferrocene imine ligands derived from allylamine and their vanadium(IV), cobalt(III), nickel(II), copper(II), zinc(II) and palladium(II) complexes. J. Mol. Struct..

[12] Khorshidifard M., Rudbari H.A., Askari B., Sahihi M., Farsani M.R., Jalilian F., Bruno G. (2015). Cobalt(II), copper(II), zinc(II) and palladium(II) Acetyl ferrocene imine complexes: Synthesis, characterization and catalytic performance in selective oxidation of sulfides using hydrogen peroxide under solvent-free conditions. Polyhedron.

[13] Menat S., Rudbari H.A., Askari B., Farsani M.R., Jalilian F., Dini G. (2016). Synthesis and characterization of insoluble cobalt(II), nickel(II), zinc(II) and palladium(II) Acetyl ferrocene imine complexes: Heterogeneous catalysts for oxidation of sulfides with hydrogen peroxide. Comptes Rendus Chim..

[14] Maurya M.R., Dhaka S., Avecilla F. (2015). Oxidation of secondary alcohols by conventional and microwave-assisted methods using molybdenum complexes of ONO donor ligands. New J. Chem..

[15] Keypour H., Shooshtari A., Rezaeivala M., Kup F.O., Rudbari H.A. (2015). Synthesis of two new N_2_O_4_ macroacyclic Acetyl ferrocene imine ligands and their mononuclear complexes: Spectral, X-ray crystal structural, antibacterial and DNA cleavage activity. Polyhedron.

[16] Niu M., Li Z., Li H., Li X., Dou J., Wang S. (2015). DNA/protein interaction, cytotoxic activity and magnetic properties of amino-alcohol Acetyl ferrocene imine derived Cu(II)/Ni(II) metal complexes: Influence of the nuclearity and metal ions. RSC Adv..

[17] Ebrahimipour S.Y., Sheikhshoaie I., Kautz A.C., Amerie M., Pasban-Aliabadi H., Rudbari H.A., Bruno G., Janiak C. (2015). Mono- and dioxido-vanadium(V) complexes of a tridentate ONO Acetyl ferrocene imine ligand: Synthesis, spectral characterization, X-ray crystal structure and anti-cancer activity. Polyhedron.

[18] Houlton A., Jasim N., Robert’s R.M.G., Silver J., Cunningham D., McArdle P., Higgins T. (1992). Molecular Materials containing Donor and Acceptor Groups. Synthesis, Structure and Spectroscopic Properties of Ferrocenyl Imine acetyl ferrocenest. J. Chem. Soc. Dalton Trans..

[19] Osborne A.G., Webba da Silva M., Hurst House M.B., Malik K.M.A., Opromolla G., Zanello P. (1996). Synthetic, structural and electrochemical studies on ferrocenylazines: Crystal structures of [4](1)(1,4-dimethyl-2,3-diazabuta-1,3-dien) ferrocenophane and [42](1,1′) bis(1,4-dimethyl-2,3-diazabuta-1,3-dien) ferrocenophane. J. Organomet. Chem..

[20] Neuse E.W., Meirim M.G., Blom N.F. (1988). Metallocene-containing platinum complexes as potential antitumor agents. 1. Dichloro(1,6-differrocenyl-2,5-diazahexane)platinum(II) and cis-dichlorobis(1-ferrocenylethylamine)platinum(II). Organometallics.

[21] Sangilipandi S., Sutradhar D., Bhattacharjee K., Kaminsky W., Joshi S.R., Chandra A.K., Rao K.M. (2016). Synthesis, structure, antibacterial studies and DFT some of some ruthenium, Cp∗ Rh, Cp∗ Ir and tricarbonylrhenium metal complexes containing 2-chloro-3-(3-(2-pyridyl) pyrazolyl) quinoxaline ligand. Inorg. Chim. Acta.

[22] Abu-Dief A.M., Abdel-Rahman L.H., Shehata M.R., Abdel-Mawgoud A.A.H. (2019). Novel azomethine Pd (II)-and VO (II)-based metallo-pharmaceuticals as anticancer, antimicrobial, and antioxidant agents: Design, structural inspection, DFT investigation, and DNA interaction. J. Phys. Org. Chem..

[23] Dhanaraj C.J., Hassan I.U., Johnson J., Joseph J., Joseyphus R.S. (2016). Synthesis, spectral characterization, DNA interaction, anticancer and molecular docking studies on some transition metal chelateswith bidentate ligand. J. Photochem. Photobiol. B Biol..

[24] National Committee for Clinical Laboratory Standards (2003). Method for Antifungal Disc Diffusion Susceptibility Testing of Yeast: Proposed Guideline M44-P.

[25] Deghadi R.G., Elsharkawy A.E., Ashmawy A.M., Mohamed G.G. (2022). Antibacterial and anticorrosion behavior of bioactive complexes of selected transition metal ions with new 2-acetylpyridine Schiff base. Appl. Organomet. Chem..

[26] Abu-Dief A.M., Abdel-Rahman L.H., Abdel-Mawgoud A.A.H. (2020). A robust in vitro anticancer, antioxidant and antimicrobial agents based on new metal-azomethine chelates incorporating Ag (I), Pd (II) and VO (II) cations: Probing the aspects of DNA interaction. Appl. Organomet. Chem..

[27] Al-Abdulkarim H.A., El-Khatib R.M., Aljohani F.S., Mahran A., Alharbi A., Mersal G.A.M., El-Metwaly N.M., Abu-Dief A.M. (2021). Optimization for synthesized quinoline-based Cr^3+^, VO^2+^, Zn^2+^ and Pd^2+^ complexes: DNA interaction, bio-logical assay and in-silico treatments for verification. J. Mol. Liq..

[28] Abdur Rauf S.A., Munawar K.Sh., Khan A., Abbasi R., Yasmeen M.R., Khan A.M., Khan A., Qureshi I.Z., Kraatz H.B. (2017). Zia-ur-Rehman Synthesis, spectroscopic characterization, DFT optimization and biological activities of Imine acetyl ferrocenes and their metal (II) complexes. J. Mol. Struct..

[29] Abu-Hussein A.A., Linert W. (2014). Synthesis, spectroscopic, coordination and biological activities of some organometallic complexes derived from thio-Acetyl ferrocene imine ligands. Spectrochim. Acta Part A.

[30] Mohamed G.G., Omar M.M., Hindy A.M. (2006). Metal chelates of Schiff Bases: Preparation, Characterization, and Biological Activity. Turk. J. Chem..

[31] Mahmoud W.H., Sayed F.N., Mohamed G.G. (2016). Synthesis, characterization and in vitro antimicrobial and anti-breast cancer activity studies of metal chelatesof novel pentadentate azo dye ligand. Appl. Organometal. Chem..

[32] Mahmoud W.H., Deghadi R.G., Mohamed G.G. (2017). Preparation, geometric structure, thermal and spectroscopic characterization of novel Acetyl ferrocene imine ligand and its metal chelates. Screening their anticancer and antimicrobial activities. J. Therm. Anal. Calorim..

[33] Aljohani E.T., Shehata M.R., Alkhatib F., Alzahrani S.O., Abu-Dief A.M. (2021). Development and structure elucidation of new VO^2+^, Mn^2+^, Zn^2+^, and Pd^2+^ complexes based on azomethine ferrocenyl ligand: DNA interaction, antimicrobial, antioxidant, anticancer activities, and molecular docking. Appl. Organomet. Chem..

[34] Yadav S., Singh R.V. (2011). Ferrocenyl-substituted Acetyl ferrocene imine complexes of boron: Synthesis, structural, physico-chemical and biochemical aspects. Spectrochim. Acta Part A.

[35] Becker A.D. (1993). Density-functional thermochemistry. III. The role of exact exchange. J. Chem. Phys..

[36] Lee C., Yang W., Parr R.G. (1998). Development of the Colle-Salvetti correlation-energy formula into a functional of the electron density. Phys. Rev. B Condens. Matter..

[37] Deghadi R.G., Elsharkawy A.E., Ashmawy A.M., Mohamed G.G. (2022). Can One Novel Series of Transition Metal Complexes of Oxy-dianiline Schiff Base Afford Advances in Both Biological Inorganic Chemistry and Materials Science?. Comments Inorg. Chem..

[38] Mahmoud W.H., Mohamed G.G., Magdy A. (2017). Preparation, characterization, biological activity evaluation, DFT calculations and molecular docking of chelates of diazoligand derived from m-phenylenediamine and p-chlorophenol. Appl. Organometal. Chem..

[39] Moustafa H., Mohamed G.G., Elramly S. (2020). Spectroscopic studies, Density Functional Theory calculations, and non-linear optical properties of binuclear Fe(III), Co(II), Ni(II), Cu(II), and Zn(II) complexes of OONN Schiff base ligand. J. Chin. Chem. Soc..

[40] Fleming I. (1976). Frontier Orbitals and Organic Chemical Reactions.

[41] Mondale S., Mandal S.M., Mondale T.K., Sinha Ch. (2017). Spectroscopic characterization, antimicrobial activity, DFT computation and docking studies of sulfonamide Imine acetyl ferrocenes. J. Mol. Struct..

[42] Mahmoud W.H., Mahmoud N.F., Mohamed G.G. (2017). Synthesis, physicochemical characterization, geometric structure and molecular docking of new biologically active ferrocene based Acetyl ferrocene imine ligand with transition metal ions. Appl. Organometal. Chem..

[43] Al-Dawood A.Y., El-Metwaly N.M., El-Ghamry H.A. (2016). Molecular docking and DFT studies on some nano-meter binuclear complexes derived from hydrazine-carbothioamide ligand, synthesis, thermal, kinetic and spectral characterization. J. Mol. Liq..

[44] Abdel Aziz A.A., Elantabli F.M., Mostafa H., El-Medani S.M. (2017). Spectroscopic, DNA Binding Ability, Biological, DFT Calculations, and Non Linear Optical Properties (NLO) of Novel Co(II), Cu(II), Zn(II), Cd(II) and Hg(II) Complexes with ONS Imine acetyl ferrocene. J. Mol. Struct..

[45] Qasem H.A., Aouad M.R., Al-Abdulkarim H.A., Al-Farraj E.S., Attar R.M., El-Metwaly N.M., Abu-Dief A.M. (2022). Tailoring of some novel bis-hydrazone metal chelates, spectral based characterization and DFT calculations for pharmaceutical applications and in-silico treatments for verification. J. Mol. Struc..

[46] Neelima M., Kavita P., Sarvesh K.S., Dinesh K. (2016). Synthesis, characterization and antimicrobial activity of Acetyl ferrocene imine Ce(III) complexes. Polyhedron.

[47] Güveli S., Özdemir N., Bal-Demirci T., Ülküseven B., Dinçer M., Andaç O. (2010). Quantum-chemical, spectroscopic and X-ray diffraction studies on nickel complex of 2-hydroxyacetophenone thiosemicarbazone with triphenylphospine. Polyhedron.

[48] Abdel-Rahman L.H., Abu-Dief A.M., Moustafa H., Abdel-Mawgoud A.A.H. (2020). Design and nonlinear optical properties (NLO) using DFT approach of new Cr (III), VO (II), and Ni (II) chelates incorporating tri-dentate imine ligand for DNA interaction, antimicrobial, anticancer activities and molecular docking studies. Arab. J. Chem..

[49] Dehkhodaei M., Khorshidifard M., Rudbari H.A., Sahihi M., Azimi Gh., Habibi N., Taheri S., Bruno G., Azadbakht R. (2017). Synthesis, characterization, crystal structure and DNA, HSA-binding studies of four Acetyl ferrocene imine complexes derived from salicylaldehyde and isopropylamine. Inorg. Chim. Acta.

[50] Shabbir M., Akhter Z., Ahmad I., Ahmed S., Bolte M., Ismail H., Mirza B. (2017). Ferrocene-based Imine acetyl ferrocenes copper (II) complexes: Synthesis, characterization, biological and electrochemical analysis. Inorg. Chim. Acta.

[51] Parsaee Z., Mohammadi K. (2017). Synthesis, characterization, nano-sized binuclear nickel complexes, DFT calculations and antibacterial evaluation of new macrocyclic Acetyl ferrocene imine compounds. J. Mol. Struct..

[52] Mahmoud W.H., Mahmoud N.F., Mohamed G.G. (2017). New nanobidentate Schiff base ligand of 2-aminophenol with 2-acetylferrocene with some lanthanide metal ions: Synthesis, characterization and Hepatitis A, B, C and breast cancer docking studies. J. Coord. Chem..

[53] Abu-Dief A.M., El-Metwaly N.M., Alzahrani S.O., Alkhatib F.M., Abualnaja M., El-Dabea T., Ali M.A.A. (2021). Synthesis and characterization of Fe (III), Pd (II) and Cu (II)-thiazole complexes; DFT, pharmacophore modeling, in-vitro assay and DNA binding studies. J. Mol. Liq..

[54] Abu-Dief A.M., Abdel-Rahman L.H., Abdelhamid A.A., Marzouk A.A., Shehata M.R., Bakheet M.A., Almaghrabi O.A., Nafady A. (2020). Synthesis and characterization of new Cr (III), Fe (III) and Cu (II) complexes incorporating multi-substituted aryl imidazole ligand: Structural, DFT, DNA binding, and biological implications. Spectrochim. Acta A.

[55] Abdel-Rahman L.H., Adam M.S., Abu-Dief A.M., Ahmed H.E., Nafady A. (2020). Non-linear optical property and biological assays of therapeutic potentials under in vitro conditions of Pd (II), Ag (I) and Cu (II) complexes of 5-diethyl amino-2-({2-[(2-hydroxy-Benzylidene)-amino]-phenylimino}-methyl)-phenol. Molecules.

[56] Munde A.S., Shelke V.A., Jadhav S.M., Kirdant A.S., Vaidya S.R., Shankar War S.G., Chondhekar T.K. (2012). Synthesis, characterization and antimicrobial activities of some transition metal chelatesof biologically active asymmetrical tetradentate ligands. Adv. Appl. Sci. Res..

[57] Abdel-Rahman L.H., Abdelhamid A.A., Abu-Dief A.M., Shehata M.R., Bakhe M.A. (2020). Facile synthesis, X-Ray structure of new multi-substituted aryl imidazole ligand, biological screening and DNA binding of its Cr (III), Fe (III) and Cu (II) coordination compounds as potential antibiotic and anticancer drugs. J. Mol. Struct..

[58] Chohan Z.H., Pervez H., Rauf A., Khan K.M., Supuran C.T. (2004). Isatin-derived Antibacterial and Antifungal Compounds and their Transition Metal Complexes. J. Enzyme Inhib. Med. Chem..

[59] Abu-Dief A.M., El-Sagher H.M., Shehata M.R. (2019). Fabrication, spectroscopic characterization, calf thymus DNA binding investigation, antioxidant and anticancer activities of some antibiotic azomethine Cu (II), Pd (II), Zn (II) and Cr (III) complexes. Appl. Organomet. Chem..

[60] Kaushik S., Rikhi M., Bhatnagar S. (2015). Docking and cytotoxicity studies of 2-vinylchromone derivatives on human breast cancer cell lines. Int. J. Pharm. Pharm. Sci..

[61] Abdelgawada M.A., Belal A., Ahmed O.M. (2013). Synthesis, molecular docking studies and cytotoxic screening of certain novel thiazolidinone derivatives substituted with benzothiazole or benzoxazole. J. Chem. Pharm. Res..

[62] Rehman W., Yasmeen R., Rahim F., Waseem M., Guod C.Y., Hassan Z., Rashid U., Ayub Kh. (2016). Synthesis biological screening andmolecular docking studies of some tin (IV) Acetyl ferrocene imine adducts. J. Photochem. Photobiol. B Biol..

