# Supplementary material for: Synthesis, Spectroscopic, Structural and Molecular Docking Studies of Some New Nano-Sized Ferrocene-Based Imine Chelates as Antimicrobial and Anticancer Agents
Source: Materials (Basel). 2022 May 20;15(10):3678. doi: 10.3390/ma15103678 (PMC9144163; doi:10.3390/ma15103678)
Supplement: Supplementary file 1 [file materials-15-03678-s001.zip › materials-1713531-supplementary.pdf]

materials

*Article*

# Synthesis, Spectroscopic, structural and molecular docking studies of some new nano-sized ferrocene based imine chelates as antimicrobial and anticancer agents

**Mai M. Khalaf[1,2*], Hany M. Abd El-Lateef[1,2], A. Alhadhrami[3], Fatma N. Sayed[4], Gehad G. Mohamed[4,5], M. Gouda[1], Saad Shaaban[1,6] and Ahmed M. Abu-Dief[2,7*]**

1 Department of Chemistry, College of Science, King Faisal University, Al-Ahsa 31982, Saudi Arabia

2 Department of Chemistry, Faculty of Science, Sohag University, Sohag 82534, Egypt

3 Department of Chemistry, Colleague of Science, Taif University, P.O. Box 11099, Taif, 21944, Saudi Arabia

4 Chemistry Department, Faculty of Science, Cairo University, Giza, 12613, Egypt

5 Nanoscience Department, Basic and Applied Sciences Institute, Egypt-Japan University of Science and Technology, New Borg El Arab, Alexandria, 21934, Egypt

6 Chemistry Department, Faculty of Science, Mansoura University, Mansoura 35516, Egypt

7 Department of Chemistry, College of Science, Taibah University, Madinah P.O. Box 344, Saudi Arabia

* Correspondence: amamohammed@taibahu.edu.sa (A. M. A-D); mmkali@kfu.edu.sa (M.M. K.)

**Instruments**

Mass spectra were recorded by the EI technique at 70 eV using an MS-5988 GS–MS Hewlett–Packard instrument at the Microanalytical Center, National Center for Research, Egypt. Molar conductivities of $10^{-3}$ M solutions of the solid complexes in ethanol were measured using a Jenway 4010 conductivity meter. Microanalyses of carbon, hydrogen and nitrogen were carried out at the Microanalytical Center, Cairo University, Egypt, using a CHNS-932 (LECO) Vario Elemental Analyzer. Analyses of the metals followed the dissolution of the solid complexes in concentrated $HNO_3$, neutralizing the diluted aqueous solutions with ammonia and titrating the metal solutions with EDTA. FT-IR spectra were recorded on a Perkin-Elmer 1650 spectrometer (4000–400 $cm^{-1}$) as KBr pellets. Electronic spectra were recorded at room temperature on a Shimadzu 3101pc spectrophotometer as solutions in ethanol. UV–Vis spectra were carried out on UV mini-1240, UV–Vis spectrophotometer, Shimadzu. The thermogravimetric analyses (TG and DTG) of the solid complexes were carried out from room temperature to 1000 $^{o}C$ using a Shimadzu TG-50H thermal analyzer. The scanning electron microscopic (SEM) image of the complexes was recorded by using SEM model Quanta 250 FEG (Field Emission Gun) attached with EDX Unit (Energy Dispersive X-ray Analyses), with accelerating voltage 30 K.V., magnification 14x up to 1000000 and resolution for Gun.1n, National Research Center, Egypt. The antimicrobial activities were carried out at the Microanalytical Center, Cairo University, Egypt. The anticancer activity was performed at the National Cancer Institute, Cancer Biology Department, Pharmacology Department, Cairo University. The optical density (O.D.) of each well was measured spectrophotometrically at 564 nm with an ELIZA microplate reader (Meter tech. R 960, USA).

**Solutions**

Stock solutions of metal complexes of $1x10^{-3}$ M were prepared by dissolving an accurately weighed amount of the complex in (1:3) ethanol: DMF. Solution of the Schiff base ligand ($1x10^{-4}$ M) and its metal complexes ($1x10^{-5}$ M) were prepared by dilution of the previous prepared stock solutions for measuring their UV–Vis spectra.

**Solution of anticancer study**

A fresh stock solution of ($1x10^{-3}$ M) of Schiff base ligand (0.0032 g/L) was prepared in the appropriate volume of ethanol (95 %). Dimethylsulphoxide (DMSO) (Sigma Chemical Co., St. Louis, MO, USA) was used in the cryopreservation of cells. RPMI-1640 medium (Sigma Chemical Co., St. Louis, MO, USA) was used. The medium was used to culture and maintain the human tumor cell line. The medium was supplied in a powder form. It was prepared as follows: 10.4 g medium was weighed, mixed with 2 g sodium bicarbonate, and distilled water was added to 1 L, and the mixture was shaken carefully until complete dissolution. The medium was then sterilized by filtration in a

Millipore bacterial filter (0.22 μm). The prepared medium was kept in a refrigerator (4 °C) and checked at regular intervals for contamination. Before use, the medium was warmed at 37 °C in a water bath and supplemented with penicillin/streptomycin and FBS. Sodium bicarbonate (Sigma Chemical Co., St. Louis, MO, USA) was used for the preparation of RPMI-1640 medium. Isotonic Trypan blue solution (0.05 %; Sigma Chemical Co., St. Louis, MO, USA) was prepared in normal saline and was used for viability counting. Fetal bovine serum (10 %; FBS) (heat inactivated at 56 °C for 30 min), 100 units/mL penicillin and 2 mg/mL streptomycin were supplied from Sigma Chemical Co., St. Louis, MO, USA and were used for the supplementation of RPMI-1640 medium prior to use. Trypsin (0.025 % (w/v); Sigma Chemical Co., St. Louis, MO, USA) was used for the harvesting of cells. Acetic acid (1 % (v/v); Sigma Chemical Co., St. Louis, MO, USA) was used for dissolving the unbound SRB dye. Sulphorhodamine-B (0.4 %; SRB) (Sigma Chemical Co., St. Louis, MO, USA) dissolved in 1 % acetic acid was used as a protein dye. A stock solution of trichloroacetic acid (TCA, 50 %, Sigma Chemical Co., St. Louis, MO, USA) was prepared and stored. Fifty microliters of the stock were added to 200 μL RPMI-1640 medium/well to yield a final concentration of 10 % used for protein precipitation. One hundred percent Isopropanol and 70 % ethanol were used. Tris base 10 mM (pH 10.5) was used for SRB dye solubilization. Tris base (121.1 g) was dissolved in 1000 mL of distilled water, and the pH was adjusted with HCl acid (2 M).

**Biological activity**

**Anti-pathogenic activity**

The Well diffusion approach was used to test in vitro antifungal and antibacterial activities of ferrocene imine ligand and its meatal chelates. DMSO has been used to prepare the stock solutions of different concentrations (μg/mL) to evaluate antimicrobial efficiency. The medium for antifungal activity was prepared by adding 65 g Sabround Dextrose Agar (SDA) in 1 L of distilled water (DW). The mixture of SDA and DW was heated with stirring to form a uniform solution. The sterilization is performed by autoclaving this solution (media) for 15 min at 121 ºC. The media were spread uniformly on sterile Petri plates having a diameter of 90 mm and kept for rest until solidify. The same procedure was followed to prepare nutrient agar (NA) medium i.e., 28 g NA in 1 L of DW to explore antibacterial activity. After solidification, four wells (4 mm diameter) were taken out through sterile cork from the solidified media. The suspension form of the tested fungi was spread over the surface and the separate wells were filled with different concentration stock solutions. Finally, the Petri plates were sealed and stored at low temperature for 2 to 3 hr. for diffusion and then incubated at room temperature (27 ºC) for 24 hr., then the zones of inhibition were evaluated. (mm).

**Optimization of anticancer study**

To assess the cytotoxicity of the prepared compounds, MCF-7 cell line was incubated in DMEM medium containing FBS (10 percent v/v), streptomycin (100 g/ml), and penicillin (100 g/ml) and maintained at 37°C in a 5 percent $CO_2$ incubator [33, 35]. Following the incubation period, serial dilutions of the compounds were added to the wells in triplicates, and the incubation period was extended for another 48 hours. Before 1 hour from the end of the incubation, 20 μL of MTT (5 g/mL in PBS) was applied to each well. The plates were shaken after the incubation time and the su-pernatant liquid was extracted. Each well received 100 μL of DMSO. After 15 minutes of room temperature incubation, the optical density (OD) was measured at 540 nm. The be-low formula was used to calculate the percentage of cells that were dead %Inhibition = {(Abscontrol – Abssample)/(Abssample )} x 100

The IC50 values of the compounds were generated from the dose-response curves and reported as the average of three independent experiments. The IC50 value is determined as the average standard deviation.

Table S1. The different optimized parameters of the free Schiff base ligand.

| HL | Bond Length | HL | Bond Length |
|---|---|---|---|
| C(1)-C(2) | 1.4479 | C(12)-H(16) | 1.0812 |
| C(1)-C(5) | 1.4516 | C(13)-C(17) | 1.4435 |
| C(1)-Fe(10) | 2.1209 | C(13)-H(18) | 1.0811 |
| C(1)-C(21) | 1.4755 | C(15)-C(17) | 1.4423 |
| C(2)-C(3) | 1.4356 | C(15)-H(19) | 1.0809 |
| C(2)-H(6) | 1.0794 | C(17)-H(20) | 1.0813 |
| C(2)-Fe(10) | 2.1212 | C(21)-N(22) | 1.3034 |
| C(3)-C(4) | 1.4452 | C(21)-C(32) | 1.5241 |
| C(3)-H(7) | 1.081 | N(22)-C(23) | 1.411 |
| C(3)-Fe(10) | 2.1261 | C(23)-C(24) | 1.4211 |
| C(4)-C(5) | 1.4385 | C(23)-C(25) | 1.417 |
| C(4)-H(8) | 1.0809 | C(24)-C(26) | 1.4059 |
| C(4)-Fe(10) | 2.1201 | C(24)-S(37) | 1.8425 |
| C(5)-H(9) | 1.0807 | C(25)-C(27) | 1.4047 |
| C(5)-Fe(10) | 2.1115 | C(25)-H(36) | 1.0873 |
| Fe(10)-C(11) | 2.119 | C(26)-C(28) | 1.4081 |
| Fe(10)-C(12) | 2.1178 | C(26)-H(29) | 1.0879 |
| Fe(10)-C(13) | 2.1211 | C(27)-C(28) | 1.4072 |
| Fe(10)-C(15) | 2.1207 | C(27)-H(30) | 1.0871 |
| Fe(10)-C(17) | 2.1227 | C(28)-H(31) | 1.0869 |
| C(11)-C(12) | 1.443 | C(32)-H(33) | 1.0923 |
| C(11)-C(13) | 1.4433 | C(32)-H(34) | 1.097 |
| C(11)-H(14) | 1.0811 | C(32)-H(35) | 1.0983 |
| C(12)-C(15) | 1.4435 | S(37)-H(38) | 1.3797 |

| HL | Bond angle | HL | Bond angle |
|---|---|---|---|
| C(2)-C(1)-C(5) | 107.5437 | C(13)-Fe(10)-C(15) | 66.8433 |
| C(2)-C(1)-C(21) | 125.2253 | Fe(10)-C(11)-H(14) | 124.8814 |
| C(5)-C(1)-C(21) | 127.2304 | C(12)-C(11)-C(13) | 107.9952 |
| Fe(10)-C(1)-C(21) | 125.4684 | C(12)-C(11)-H(14) | 125.9952 |
| C(1)-C(2)-C(3) | 108.1748 | C(13)-C(11)-H(14) | 126.0066 |
| C(1)-C(2)-H(6) | 124.2325 | Fe(10)-C(12)-H(16) | 124.7961 |
| C(3)-C(2)-H(6) | 127.5857 | C(11)-C(12)-C(15) | 108.0573 |
| H(6)-C(2)-Fe(10) | 125.9356 | C(11)-C(12)-H(16) | 125.9565 |
| C(2)-C(3)-C(4) | 108.2076 | C(15)-C(12)-H(16) | 125.9831 |
| C(2)-C(3)-H(7) | 125.9467 | Fe(10)-C(13)-H(18) | 125.5184 |
| C(4)-C(3)-H(7) | 125.844 | C(11)-C(13)-C(17) | 107.9221 |
| H(7)-C(3)-Fe(10) | 125.3745 | C(11)-C(13)-H(18) | 125.9476 |
| C(3)-C(4)-C(5) | 108.0273 | C(17)-C(13)-H(18) | 126.1299 |

| | | | |
|---|---|---|---|
| C(3)-C(4)-H(8) | 126.0682 | Fe(10)-C(15)-H(19) | 124.9823 |
| C(5)-C(4)-H(8) | 125.9045 | C(12)-C(15)-C(17) | 107.9111 |
| H(8)-C(4)-Fe(10) | 125.3766 | C(12)-C(15)-H(19) | 126.1309 |
| C(1)-C(5)-C(4) | 108.043 | C(17)-C(15)-H(19) | 125.9562 |
| C(1)-C(5)-H(9) | 126.3247 | Fe(10)-C(17)-H(20) | 125.1758 |
| C(4)-C(5)-H(9) | 125.6309 | C(13)-C(17)-C(15) | 108.1141 |
| H(9)-C(5)-Fe(10) | 125.2221 | C(13)-C(17)-H(20) | 126.1664 |
| C(1)-Fe(10)-C(3) | 66.7205 | C(15)-C(17)-H(20) | 125.7179 |
| C(1)-Fe(10)-C(4) | 66.9388 | C(1)-C(21)-N(22) | 117.4401 |
| C(1)-Fe(10)-C(11) | 160.7728 | C(1)-C(21)-C(32) | 117.1205 |
| C(1)-Fe(10)-C(13) | 125.631 | N(22)-C(21)-C(32) | 125.386 |
| C(1)-Fe(10)-C(15) | 123.9885 | N(22)-C(23)-C(24) | 118.4337 |
| C(1)-Fe(10)-C(17) | 110.2405 | N(22)-C(23)-C(25) | 122.7639 |
| C(2)-Fe(10)-C(4) | 66.7664 | C(24)-C(23)-C(25) | 118.5067 |
| C(2)-Fe(10)-C(5) | 67.0898 | C(23)-C(24)-C(26) | 120.2396 |
| C(2)-Fe(10)-C(11) | 157.6029 | C(23)-C(24)-S(37) | 116.9546 |
| C(2)-Fe(10)-C(12) | 122.7853 | C(26)-C(24)-S(37) | 122.8042 |
| C(2)-Fe(10)-C(13) | 161.0694 | C(23)-C(25)-C(27) | 121.0734 |
| C(2)-Fe(10)-C(15) | 109.0414 | C(23)-C(25)-H(36) | 118.7598 |
| C(2)-Fe(10)-C(17) | 125.2372 | C(27)-C(25)-H(36) | 120.1411 |
| C(3)-Fe(10)-C(5) | 66.8214 | C(24)-C(26)-C(28) | 120.5349 |
| C(3)-Fe(10)-C(11) | 122.8689 | C(24)-C(26)-H(29) | 120.0204 |
| C(3)-Fe(10)-C(12) | 108.2379 | C(28)-C(26)-H(29) | 119.442 |
| C(3)-Fe(10)-C(15) | 123.9178 | C(25)-C(27)-C(28) | 119.8799 |
| C(3)-Fe(10)-C(17) | 159.8668 | C(25)-C(27)-H(30) | 119.8346 |
| C(4)-Fe(10)-C(11) | 108.6956 | C(28)-C(27)-H(30) | 120.2851 |
| C(4)-Fe(10)-C(12) | 123.6684 | C(26)-C(28)-C(27) | 119.7465 |
| C(4)-Fe(10)-C(13) | 123.8068 | C(26)-C(28)-H(31) | 119.7435 |
| C(4)-Fe(10)-C(15) | 159.1627 | C(27)-C(28)-H(31) | 120.5073 |
| C(5)-Fe(10)-C(11) | 124.5277 | C(21)-C(32)-H(33) | 111.7115 |
| C(5)-Fe(10)-C(12) | 159.4475 | C(21)-C(32)-H(34) | 109.7076 |
| C(5)-Fe(10)-C(13) | 109.8714 | C(21)-C(32)-H(35) | 110.8013 |
| C(5)-Fe(10)-C(17) | 124.7405 | H(33)-C(32)-H(34) | 108.6066 |
| C(11)-Fe(10)-C(15) | 66.872 | H(33)-C(32)-H(35) | 108.6378 |
| C(11)-Fe(10)-C(17) | 66.7798 | H(34)-C(32)-H(35) | 107.2457 |
| C(12)-Fe(10)-C(13) | 66.853 | C(24)-S(37)-H(38) | 95.5784 |
| C(12)-Fe(10)-C(17) | 66.7686 | | |

Table S2. Biological activity of organometallic Schiff base (HL) and its metal complexes.

| | Inhibition zone (mm/mg sample) | | | | | |
|---|---|---|---|---|---|---|
| **Compound** | Fungi | | Gram positive bacteria | | Gram negative bacteria | |
| | *A. fumigatus* | *C. albicans* | *S. aureus* | *B. subtilis* | *S. typhimurium* | *E. coli* |
| **HL** | 18 | 16 | NA | NA | NA | 19 |
| $[Cr(HL)(H_2O)_3Cl]Cl_2.2H_2O$ | 17 | 13 | NA | NA | 14 | 18 |
| $[Mn(HL)(H_2O)_4]Cl_2.2H_2O$ | NA | 10 | NA | NA | NA | 12 |
| $[Fe(HL)(H_2O)_3Cl]Cl_2.3H_2O$ | NA | NA | 18 | 16 | 15 | 16 |
| $[Co(HL)(H_2O)_3Cl]Cl.2H_2O$ | NA | NA | NA | NA | NA | NA |
| $[Ni(HL)(H_2O)_3Cl]Cl.2H_2O$ | NA | NA | 16 | 20 | 13 | 21 |
| **Control** | *Ketokenazole* | | *Gentamycin* | | *Gentamycin* | |
| | 17 | 20 | 24 | 26 | 17 | 30 |

Table S3. Anticancer activity of Schiff base ligand and its metal complexes.

| **Complex** | **Surviving fraction (MCF7)** | | | | | | |
|---|---|---|---|---|---|---|---|
| | **Concn. (µg/ ml)** | **0.0** | **5.0** | **12.5** | **25.0** | **50.0** | **$IC_{50}$ (µIC50g/ ml)** |
| $[Mn(HL)(H_2O)_4]Cl_2.2H_2O$ | | 1.00 | 0.82 | 0.73 | 0.64 | 0.55 | |
| $[Cu(HL)(H_2O)_3Cl]Cl.H_2O$ | | 1.00 | 0.87 | 0.77 | 0.71 | 0.64 | |
| $[Zn(HL)Cl_2].2H_2O$ | | 1.00 | 1.00 | 0.74 | 0.68 | 0.65 | |
| $[Cd(HL)(H_2O)_2Cl_2]$ | | 1.00 | 0.50 | 0.31 | 0.33 | 0.39 | 5.57 |

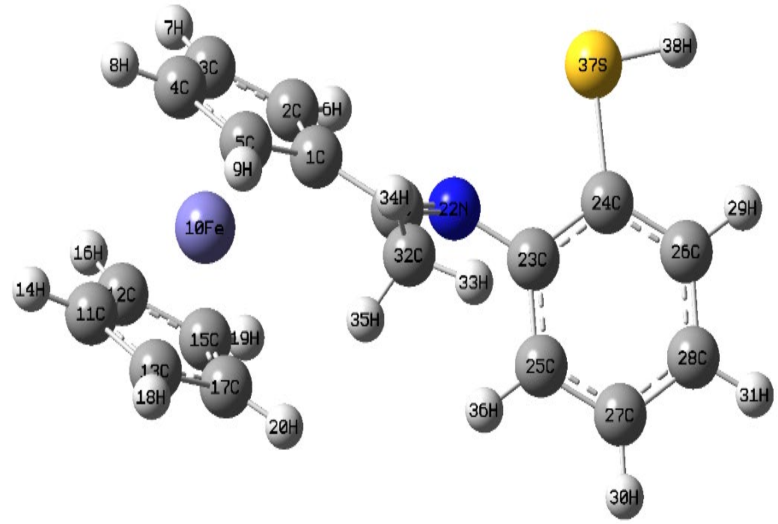


Figure S1. The optimized structure of the newly synthesized acetyl ferrocene imine ligand.

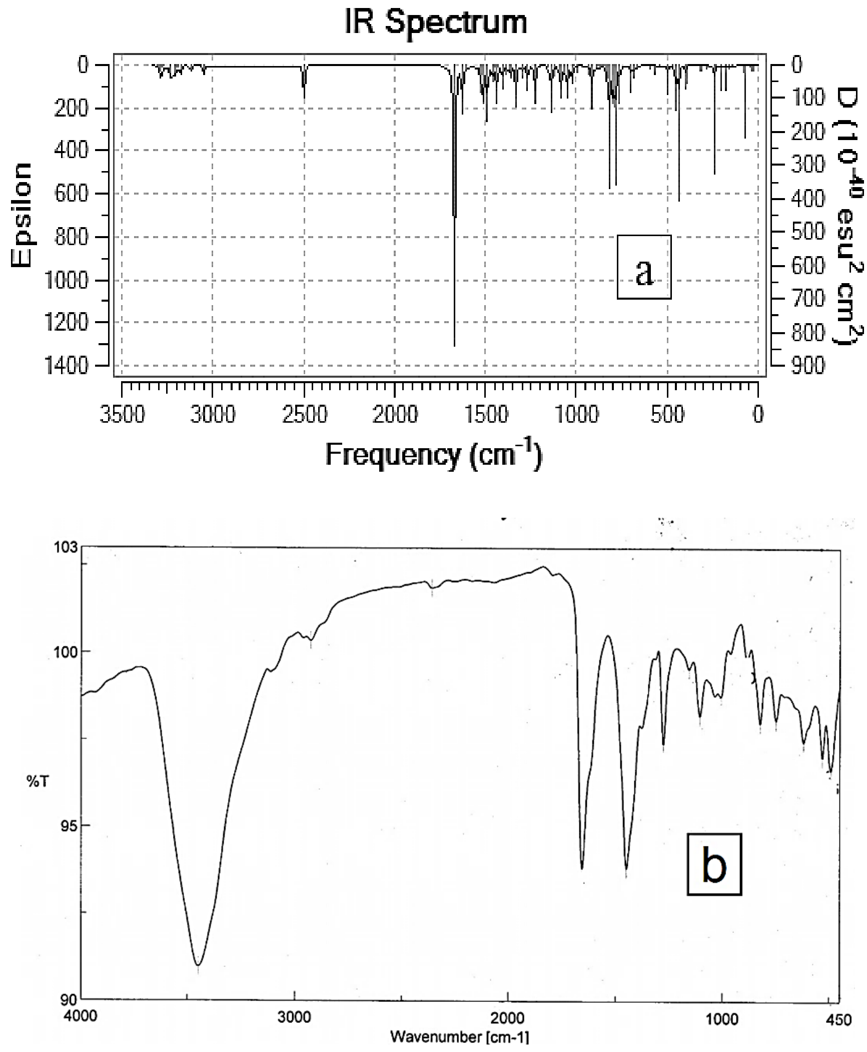


Figure S2. IR spectra of the free ligand (a) theoretical and (b) experimental.

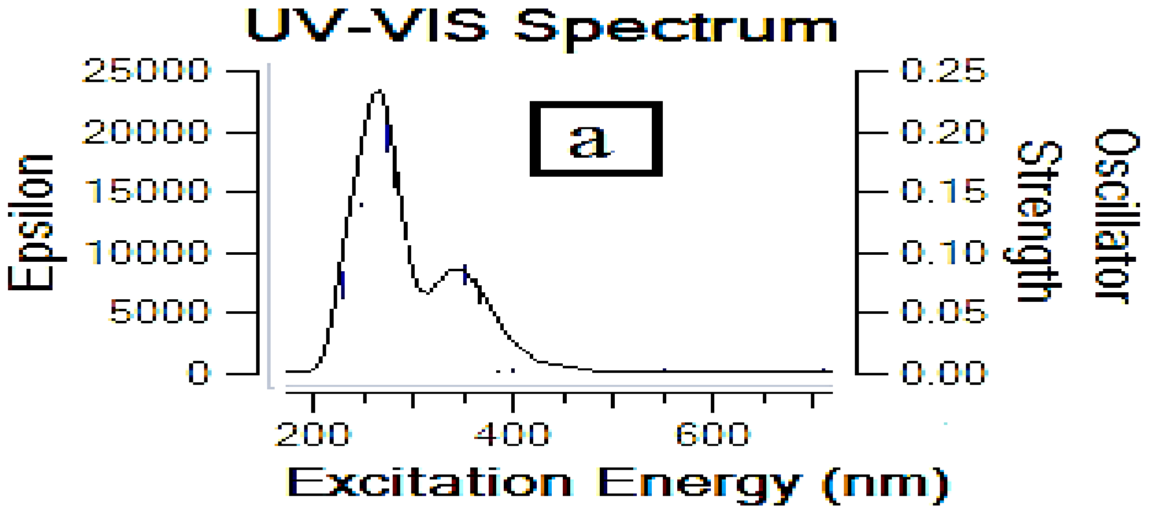


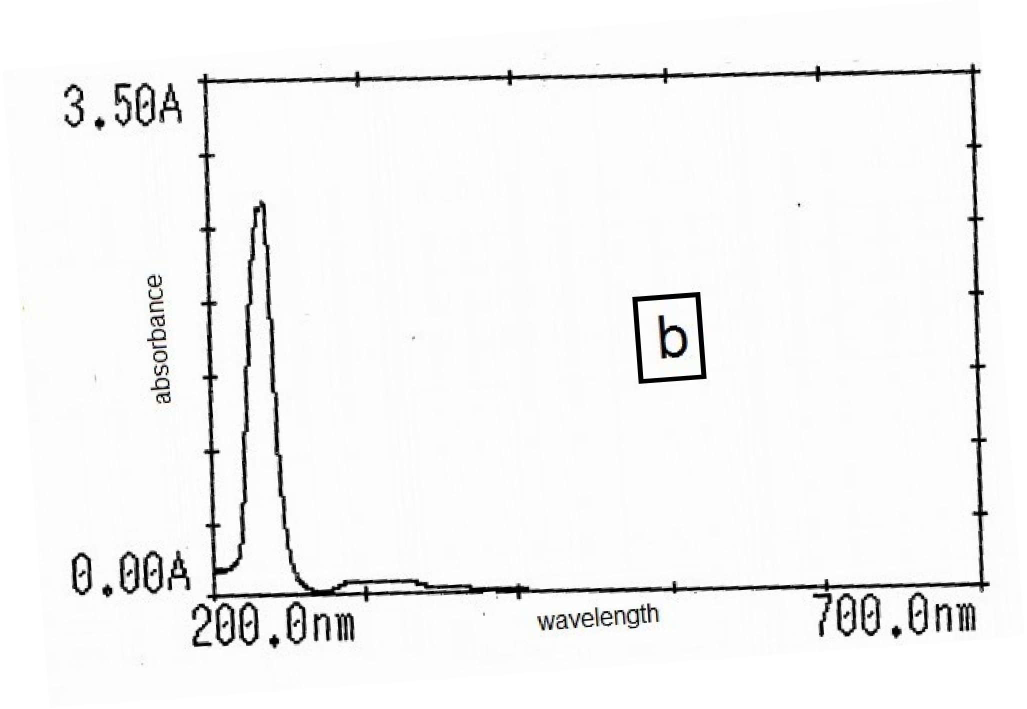


Figure S3. UV-Vis spectra of HL (a) theoretical and (b) experimental.

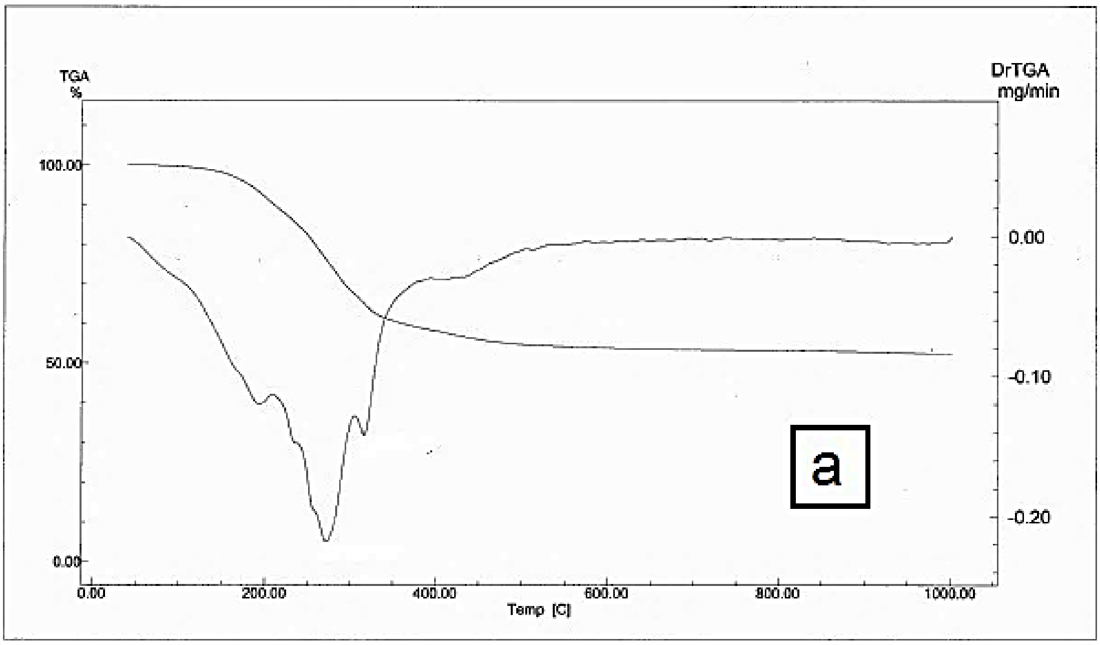
TGA
%
DrTGA
mg/min
100.00
50.00
0.00
0.00
-0.10
-0.20
0.00
200.00
400.00
600.00
800.00
1000.00
Temp [C]
a

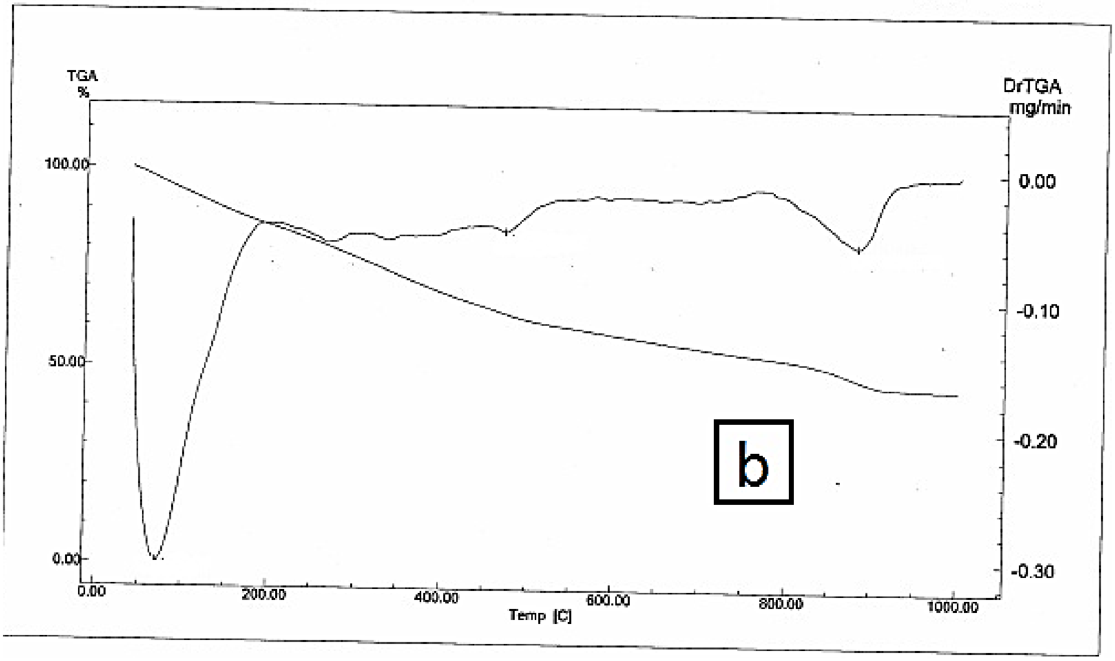
TGA
%
DrTGA
mg/min
100.00
50.00
0.00
0.00
-0.10
-0.20
-0.30
0.00
200.00
400.00
600.00
800.00
1000.00
Temp [C]
b

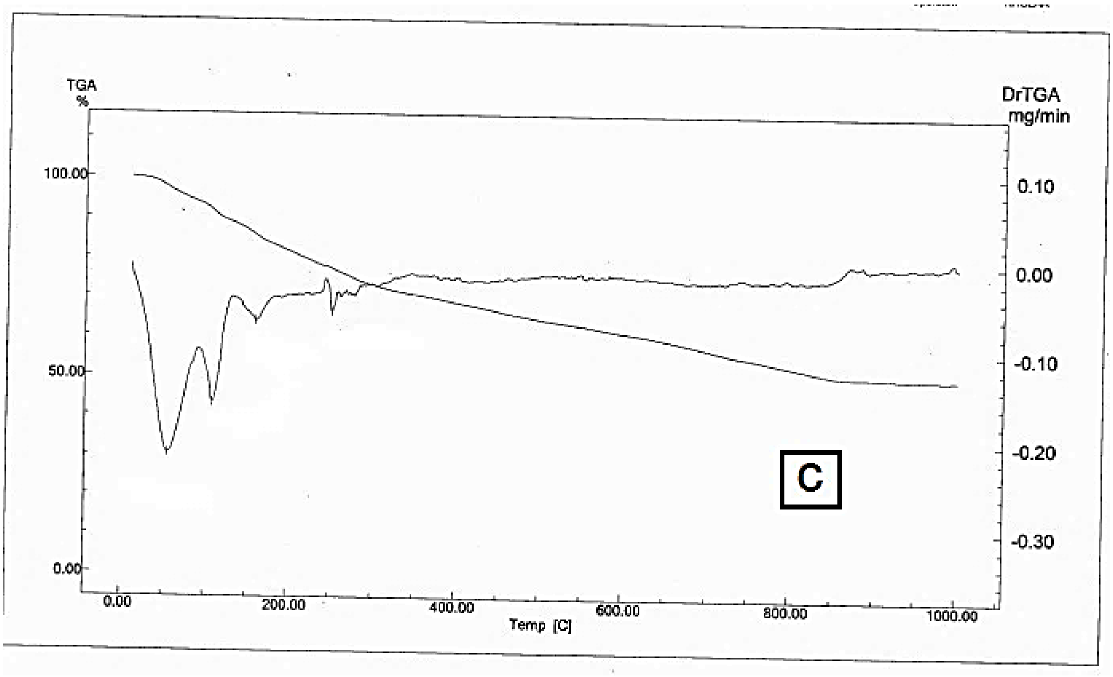
TGA
%
DrTGA
mg/min
100.00
50.00
0.00
0.10
0.00
-0.10
-0.20
-0.30
0.00
200.00
400.00
600.00
800.00
1000.00
Temp [C]
c

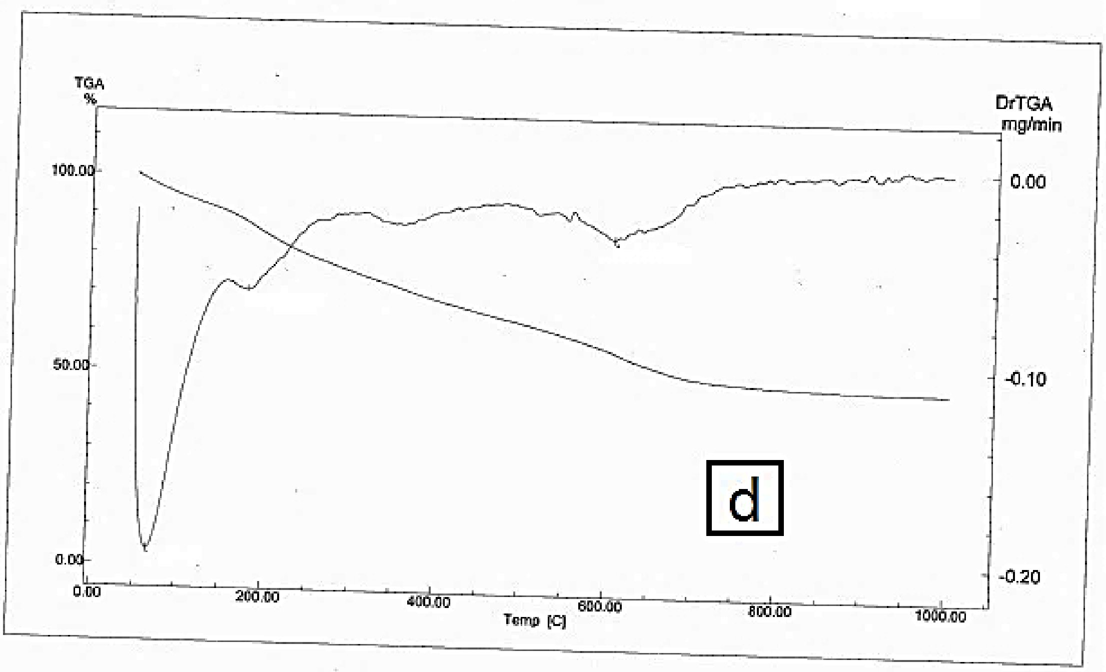
TGA
%
DrTGA
mg/min
100.00
50.00
0.00
0.00
-0.10
-0.20
0.00
200.00
400.00
600.00
800.00
1000.00
Temp [C]
d

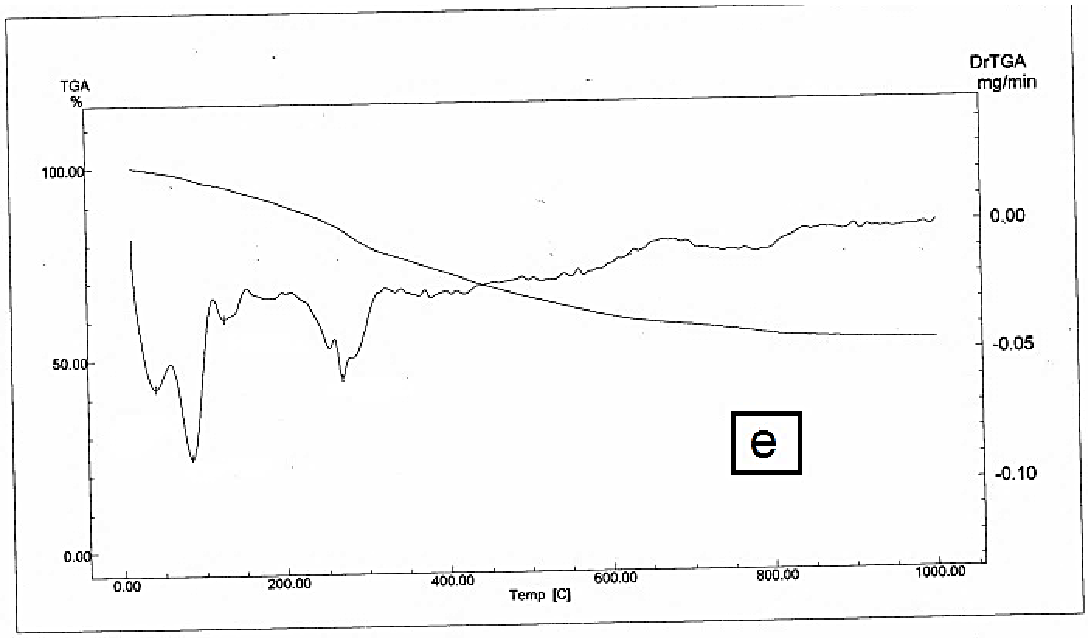
TGA
%
100.00
50.00
0.00
DrTGA
mg/min
0.00
-0.05
-0.10
0.00
200.00
400.00
600.00
800.00
1000.00
Temp [C]
e

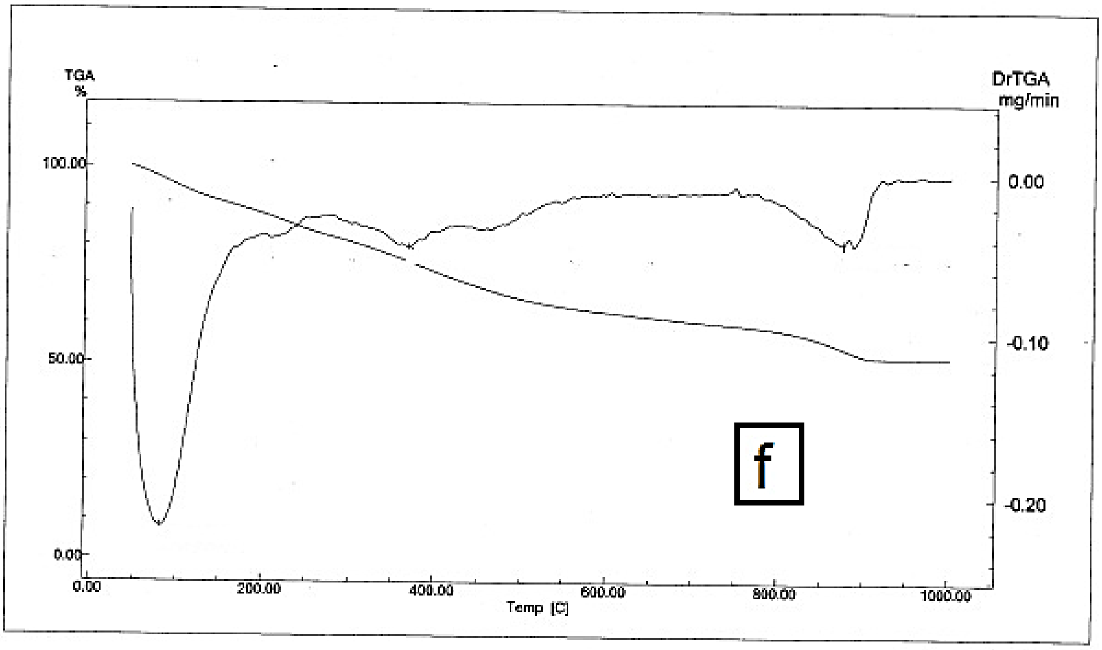
TGA
%
100.00
50.00
0.00
DrTGA
mg/min
0.00
-0.10
-0.20
0.00
200.00
400.00
600.00
800.00
1000.00
Temp [C]
f

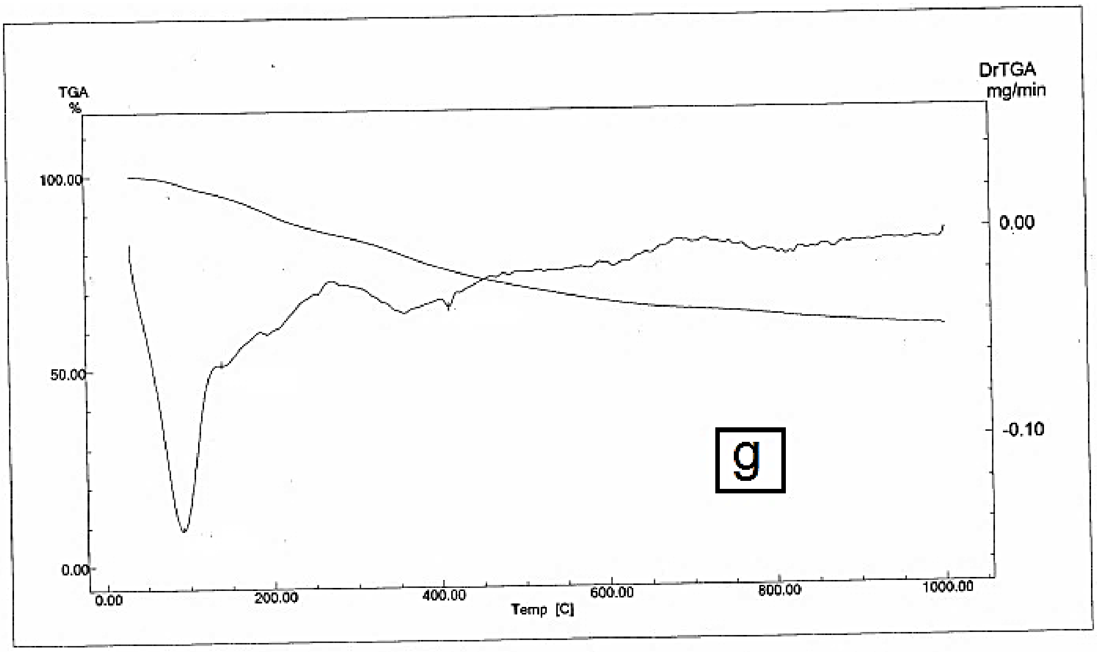

TGA
%
DrTGA
mg/min
100.00
50.00
0.00
0.00
-0.10
0.00
200.00
400.00
600.00
800.00
1000.00
Temp [C]
g


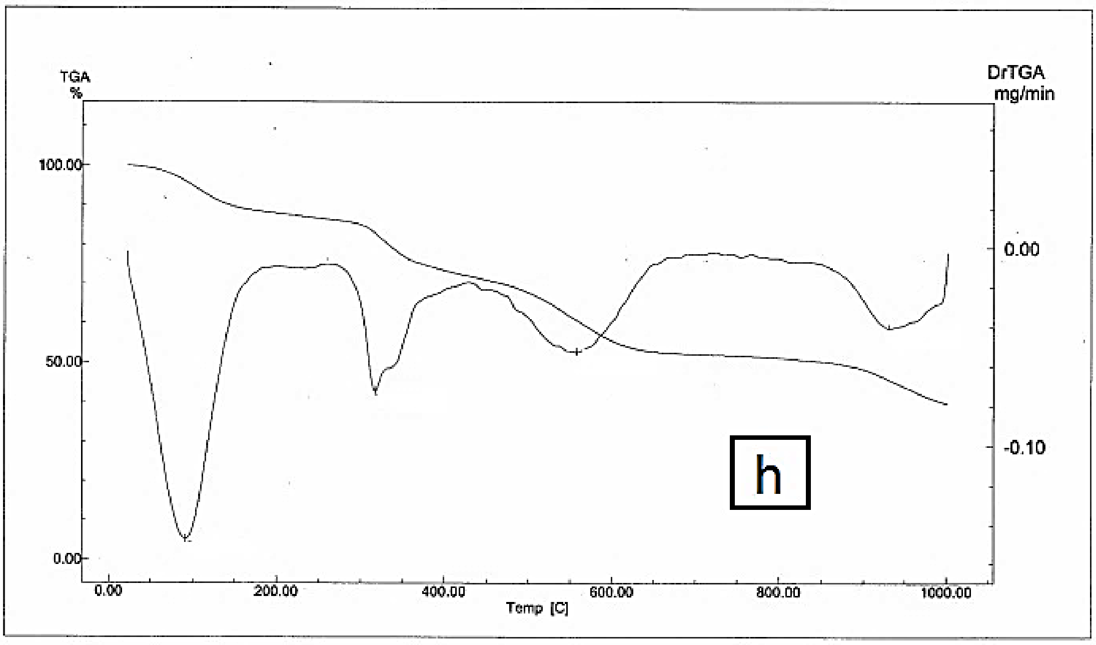

TGA
%
DrTGA
mg/min
100.00
50.00
0.00
0.00
-0.10
0.00
200.00
400.00
600.00
800.00
1000.00
Temp [C]
h

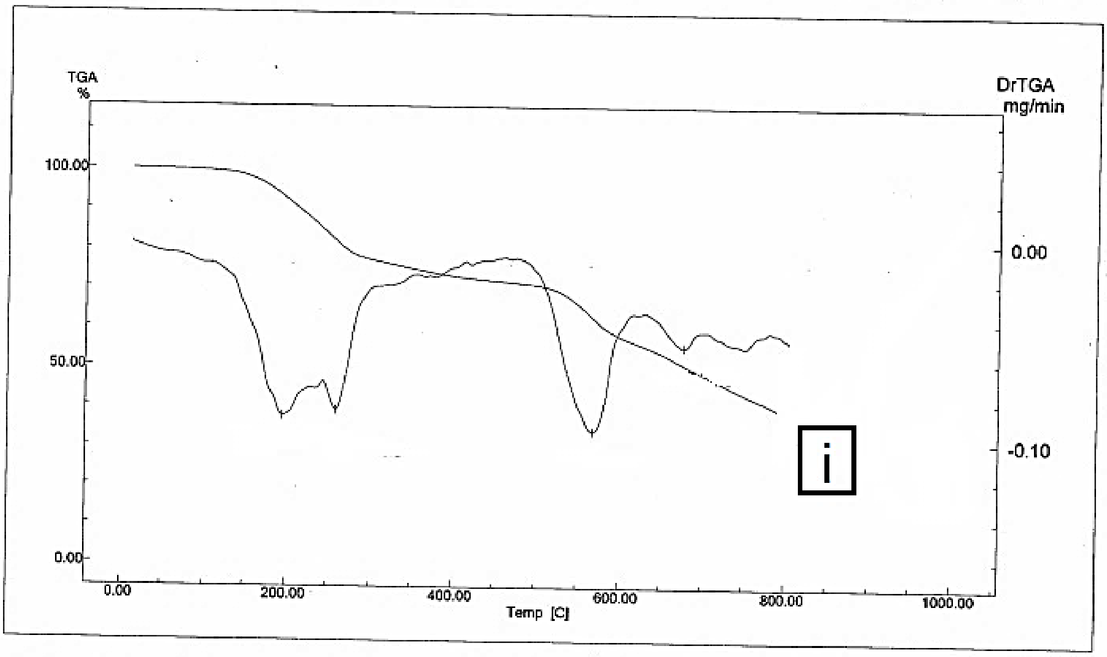


Figure S4. Thermograms of (a) HL, (b) HL-Cr(III), (c) HL-Mn(II), (d) HL-Fe(III), (e) HL-Co(II), (f) HL-Ni(II), (g) HL-Cu(II), (h) HL-Zn(II) and (i) HL-Cd(II) complexes.

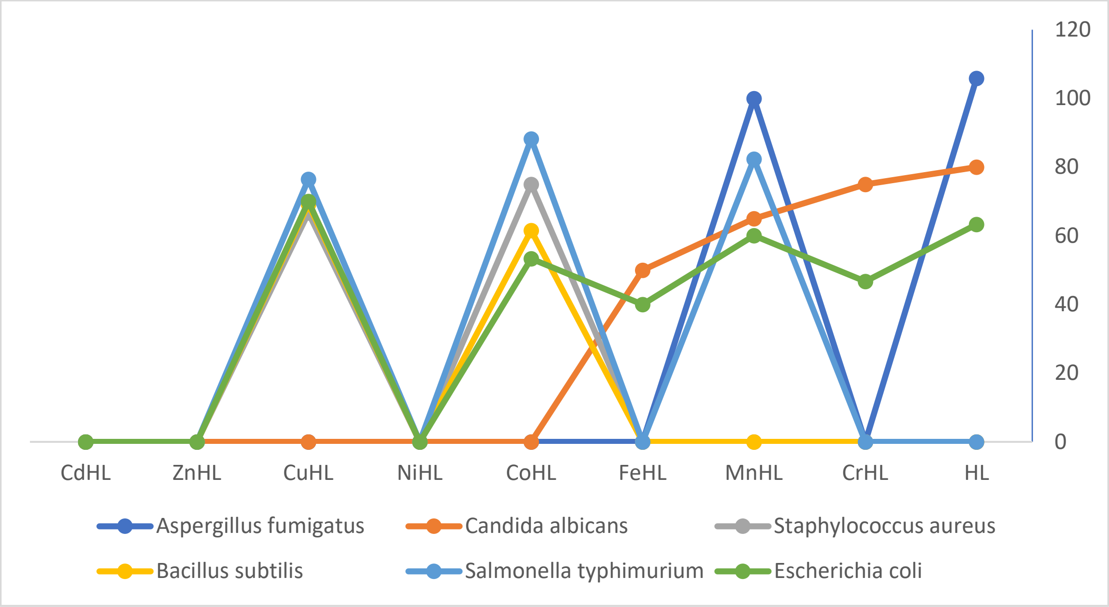


Figure S5. Activity indexes of HL and its metal complexes against tested microorganisms
